# Using contrast patterns between true complexes and random subgraphs in PPI networks to predict unknown protein complexes

**DOI:** 10.1038/srep21223

**Published:** 2016-02-12

**Authors:** Quanzhong Liu, Jiangning Song, Jinyan Li

**Affiliations:** 1 College of Information Engineering, Northwest A&F University, Yangling 712100, China; 2 Monash Bioinformatics Platform and Department of Biochemistry and Molecular Biology, Monash University, Melbourne, VIC 3800, Australia; 3 Monash Centre for Data Science, Faculty of Information Technology, Monash University, Melbourne, VIC 3800, Australia; 4 National Engineering Laboratory for Industrial Enzymes and Key Laboratory of Systems Microbial Biotechnology, Tianjin Institute of Industrial Biotechnology, Chinese Academy of Sciences, Tianjin 300308, China; 5 Advanced Analytics Institute and Centre for Health Technologies, Faculty of Engineering and IT, University of Technology Sydney, 81 Broadway, NSW 2007, Australia

## Abstract

Most protein complex detection methods utilize unsupervised techniques to cluster densely connected nodes in a protein-protein interaction (PPI) network, in spite of the fact that many true complexes are not dense subgraphs. Supervised methods have been proposed recently, but they do not answer why a group of proteins are predicted as a complex, and they have not investigated how to detect new complexes of one species by training the model on the PPI data of another species. We propose a novel supervised method to address these issues. The key idea is to discover emerging patterns (EPs), a type of contrast pattern, which can clearly distinguish true complexes from random subgraphs in a PPI network. An integrative score of EPs is defined to measure how likely a subgraph of proteins can form a complex. New complexes thus can grow from our seed proteins by iteratively updating this score. The performance of our method is tested on eight benchmark PPI datasets and compared with seven unsupervised methods, two supervised and one semi-supervised methods under five standards to assess the quality of the predicted complexes. The results show that in most cases our method achieved a better performance, sometimes significantly.

A protein complex is a group of proteins that interact one another for specific biological activities^1^. Identification of protein complexes is important for predicting protein functions^2^, disease genes^3^,^4^, phenotypic effects of genetic mutations^5^, and drug-disease associations^6^. It not only helps to characterize and understand certain biological processes^7^, also improves our understanding on human diseases^8^. Laboratory-based techniques have been developed to identify complexes. However, laboratory-based techniques are low in efficiency; and the identified complexes are usually incomplete^9^.

Over the last decade, more than 20 computational methods have been proposed as complementary tools to laboratory-based techniques for detecting protein complexes from protein-protein interaction (PPI) networks. By these methods, a PPI network is represented as a undirected graph, where the nodes stand for proteins and the edges stand for protein interactions. Given a PPI network, these methods search for protein complexes by detecting densely connected subgraphs in the network. These clustering methods can be roughly grouped into two categories^10^. The first category includes those methods solely based on PPI network topological properties, such as method MCODE (molecular complex detection)^11^, MCL (Markov cluster)^12^, CMC (clustering based on maximal cliques)^13^, CFinder^14^,^15^, ClusterONE (clustering with overlapping neighborhood expansion)^16^, RRW (repeated random walks)^17^, and an ensemble clustering method^18^. The second category includes those based on PPI network topological properties and some additional biological insights, such as a core-attachment structure focused method^19^,^20^, a restricted neighborhood search clustering algorithm (RNSC)^21^, PCIA^22^ which incorporates attribute information of the proteins in the networks, and DCAFP^9^ which makes use of functional information.

However, true complexes are not always dense subgraphs. Sometimes they can be very sparse. Figure 1 shows two sparse complexes from the the Munich Information Center for Protein Sequence (MIPS) catalogue complex database^23^. Thus, the density of a subgraph is not the only property that is useful for predicting whether the subgraph is a complex or not.

Supervised learning methods have been recently developed to detect complexes by combining other informative properties of true complexes. For example, SCI-BN^24^ is a supervised learning method which uses a Bayesian network model to learn the characteristics of true complexes. RM^25^ is a regression model for protein complex prediction. NN^26^ is a semi-supervised method taking a neural network framework. A common issue of these supervised or semi-supervised learning methods is that they cannot easily answer why a group of proteins is predicted as a complex. Moreover, they have not considered to predict new complexes of one species by training the model on the true complexes of another species. In fact, many informative properties such as degree statistics, clustering coefficient, topological coefficients and eigen values of subgraphs can be used to derive conjunctive patterns, which might be more effective than the only density feature, or the Bayesian network, regression or the neural network model, for distinguishing true complexes from random subgraphs (see our case studies).

Emerging patterns (EPs)^27^ are such type of conjunctive patterns that contrast sharply between different classes of data. For example, pattern {*meanClusteringCoeff* ≤ 0.3, 1.0 < *varDegreeCorrelation* ≤ 2.80} is an emerging pattern in the Collins^28^ PPI network. It states that if the average clustering coefficient of a subgraph is less than or equal to 0.3 *and* the degree correlation variance is between 1.0 and 2.80, then 98.2% of non-complexes (random subgraphs) exhibit this pattern, while only 6% of true complex subgraphs contain this pattern. If a new subgraph contains this EP, then it is much more likely to be a non-complex instead of a new complex. We can see that the characteristics of complexes involved in an EP is not restricted to the density property only. Rather, EPs combine novel properties of complexes to answer why a subgraph can be predicted as a complex or not. Usually, we group a set of EPs to make the prediction more reliable. Table 1 lists several other examples of EPs from the Collins PPI network.

EPs have been previously applied to deal with gene expression bioinformatics problems^29^,^30^,^31^,^32^. This work is the first time that EPs are exploited to address the complex prediction problem given a PPI network containing some true complexes. Our EP-based method consists of three main steps. The first step is to construct a feature vector to describe the key properties of the subgraphs of true complexes (positive class) in the PPI network as well as those of random non-complexes subgraphs (negative class). The second step is to discover EPs by contrasting the positive and negative classes of data. At the third step, we define an EP-based clustering score and propose a search algorithm to identify protein complexes (including overlapping complexes) from the PPI network. Our method is named ClusterEPs.

The prediction performance of ClusterEPs is compared with supervised, semi-supervised and unsupervised learning methods. Comparing with the two supervised learning methods SCI-BN^24^ and RM^25^, and the semi-supervised learning method NN^26^, ClusterEPs achieved a much better performance (precision, recall and F1) on the commonly used DIP network^33^.

As the unsupervised approach has been intensively explored in the literature, we choose to compare ClusterEPs with seven representative clustering methods. The evaluation is on five large-scale yeast PPI datasets under three standard scores, and the quality of predicted complexes is assessed with reference to the true complexes from the MIPS complex catalogue database^23^ and the Saccharomyces Genome Database (SGD)^34^. The three standard scores are: (i) the fraction of matched complexes^16^, (ii) the geometric accuracy measure^35^, and (iii) the maximum matching ratio^16^ derived from a maximal one-to-one mapping between the predicted and true complexes. Their sum is termed *composite score*, which is a more comprehensive performance measurement widely used in the field. These unsupervised clustering methods under our comparison are: MCODE^11^, MCL^12^, CMC^13^, CFinder^14^,^15^, ClusterONE^16^, RRW^17^, and RNSC^21^. Comparative experiments show that ClusterEPs can achieve a higher score of the maximum matching ratio than all the seven literature methods on all the five datasets, and a higher composite score than six of the seven methods on almost all the datasets.

Detection of new human complexes through the model learned from the complexes of yeast PPI networks is a novel attempt by this study. We trained our prediction model using the complexes from the six yeast PPI networks, and made predictions to discover new human complexes from human PPI networks. Again, ClusterEPs achieved a better performance in comparison with the other prediction methods.

Case studies are also performed to further illustrate the detailed predictive capability of ClusterEPs. It is observed that only ClusterEPs can make a complete and accurate prediction for the challenging case of the RNA polymerase I complex that consists of 14 proteins. ClusterEPs is also able to detect the RecQ helicase-Topo III complex, a small complex of only three proteins. It is highlighted here, because it is a not-well-separated subgraph, connecting to a large number of proteins outside the complex. GO analysis on the novel protein complexes predicted by our method suggests that there are strong evidences to support our prediction for these currently unknown complexes.

The algorithm has been implemented and a software version of ClusterEPs is downloadable at website http://lightning.med.monash.edu/ClusterEPs/. This website also provides a hyperlink pointing to a web server for a wider research community to use this prediction method.

## Results

This section is organized into five parts. The first part presents our comparison results with the two supervised learning methods: the Bayesian network (SCI-BN) model^24^ and the regression model (RM)^25^, and the semi-supervised learning method^26^. The second part reports our comparison results with the seven unsupervised clustering methods. The third part describes the results on the prediction of new human protein complexes using the model constructed from yeast PPI data. The fourth and fifth parts present detailed case studies and GO analysis results on the novel protein complexes predicted by our method.

### Performance comparison with supervised or semi-supervised learning methods

SCI-BN^24^ is the first supervised learning method for protein complex prediction. Yu *et al.* proposed another supervised learning method RM^25^. As the SCI-BN program is not available, Yu *et al.* used the published results to compare the performance between RM and SCI-BN. Unfortunately, the RM program is also not available to this work, we decided to compare their published results with ours.

SCI-BN and RM used two independent sets of true complexes as the positive class of data. One is the MIPS^23^ protein complex catalog, while the other one is the core set TAP06 of protein complexes from a TAP-MS experiment^36^. Taking the same way as SCI-BN and RM did, we filtered out those complexes composed of a single or a pair of proteins from the two sets of true complexes. We note that some complexes in MIPS are duplicate. Hence, our method also removed those duplicate complexes. There are 195 complexes remained in MIPS and 193 complexes remained in TAP06.

The PPI data set for the test is the well-known DIP data set^33^. A SVM-based preprocessing step was used by SCI-BN to remove all edges having a weight below 1.0. The preprocessing step by RM is to remove those interactions with a GO similarity less than 0.9. Because these two preprocessing methods are not available, we chose the TCSS (topological clustering semantic similarity) method^37^ to preprocess DIP. The semantic similarity scores between two interacting proteins compose of a biological process (BP) score, a cellular component (CC) score, and a molecular function (MF) score. We removed those interactions from DIP with a BP score less than 0.5.

We conducted experiments using MIPS as the positive training set and TAP06 as the test set and vice versa. There are a total of 1579 proteins from the complexes stored in MIPS and TAP06. Taking the same way as SCI-BN and RM did, we first extracted a PPI graph containing these proteins and their interactions from the DIP network, and then tested ClusterEPs on this PPI graph. Same measures including Precision, Recall and F1 (see Methods) were then applied to assess the performance. The results are shown in Table 2. As can be seen, when TAP06 was used as the training set and MIPS as the test set, the F1 measure of ClusterEPs was 32.2 percentage points higher than that of SCI-BN, and 12.8 percentage points higher than that of RM. When MIPS was considered as the training set and TAP06 as the test set, the F1 measure of ClusterEPs was 14.8 percentage points higher than that of SCI-BN, and 10 percentage points higher than that of RM.

NN^26^ is a semi-supervised learning model for protein complex prediction. Its performance was evaluated by using MIPS as both training set and test set. We tested ClusterEPs under this setting and the prediction performance is shown in Table 3. As can be seen, the F1 measure of ClusterEPs was 29.9 percentage points higher than that of NN, and significantly better than those of SCI-BN and RM.

### Performance comparison with unsupervised methods

Recently, Nepusz *et al.*^16^ proposed an unsupervised method named ClusterONE, and conducted a wide range of comparative studies with a group of unsupervised clustering methods on five yeast PPI networks. This group of state-of-the-art unsupervised methods include: CFinder^14^,^15^, CMC^13^, MCODE^11^, MCL^12^, RNSC^21^, RRW^17^, and ClusterONE^16^. The five yeast PPI networks are the Gavin dataset^36^, the Krogan core dataset^38^, the Krogan extended dataset^38^, the Collins dataset^28^, and BioGRID^39^. The performance evaluation was conducted under three measures (see Methods): the fraction of matched complexes (Frac), the geometric accuracy (Acc) and the maximum matching ratio (MMR), and two gold reference complex sets were used in the assessment: MIPS^23^ and SGD^34^. In line with Nepusz *et al.*^16^, we tested the performance of ClusterEPs on the same five yeast PPI networks, and compared with the above seven unsupervised clustering methods. The quality of the predicted complexes was also assessed using MIPS and SGD under the same performance measures. All parameters of the seven comparing algorithms on every PPI dataset were the same as those used in the corresponding literature^16^ for a fair comparison.

ClusterEPs was first tested on the five yeast PPI networks when SGD was applied as the positive training data. Table 4 shows the comparative performance of the eight algorithms using the MIPS standard complexes as the test set. Then, ClusterEPs was tested on these PPI networks when MIPS is used as the positive training set. Table 5 shows the comparison results when the SGD standard complexes were considered as the test set. “N/A” for the CFinder algorithm on the BioGRID dataset denotes that CFinder did not output any result within 24 hours. “N/A” for the CMC algorithm on the BioGRID dataset denotes that CMC generated a prohibitively large number of clusters (more than 6000)^16^. We can see that ClusterEPs achieved the highest MMR score on all the PPI datasets. That is, the complexes identified by ClusterEPs from the five PPI datasets had better one-to-one mapping with the reference complexes from both MIPS and SGD. For the composite scores, ClusterEPs achieved the highest scores on the Gavin, Krogan extended and BioGRID datasets (column 7 in Tables 4 and 5).

It is noteworthy that none of the eight algorithms achieved an overall best performance across all the datasets. However, except ClusterONE, our ClusterEPs algorithm achieved a consistently better composite score than the other six algorithms on four datasets. As the negative data (random subgraphs) were generated by randomly selecting nodes in the PPI networks, we ran ClusterEPs 20 times on two personal computers to calculate the average performance. The standard deviation of the 20 runs was very small for each assessment score (Supplementary Tables 17–26).

### Detection of human protein complexes through prediction model constructed on yeast protein complex data

This work was also aimed to detect novel human protein complexes from human PPI networks through a prediction model constructed using yeast complexes and PPI data. Yeast PPI networks and complexes have been deeply explored by many research teams. Our idea is to learn the basic principles of PPI networks and characteristics of protein complexes from yeast PPI and complex data, and then apply the constructed prediction model to human PPI data to transfer the basic principles for the prediction of novel human protein complexes which has been less intensively studied recently.

We used all the true yeast complexes from MIPS and SGD (MIPS + SGD) as the positive training data. The negative data were randomly generated from individual or all-combined of the six yeast PPI networks. Then emerging patterns were discovered from these positive and negative data. The prediction model was then applied to the human PPI network named HPRD (the Human Protein Reference Database Release 9)^40^ to detect new human complexes. The all-combined PPI dataset of the six yeast PPI networks contains 77051 distinct interactions after the removal of duplicate interactions. This combined dataset is named CombinedYeast. HPRD contains 37080 interactions, encompassing a high proportion of human PPIs present in the other literature databases^41^. TCSS^37^ was used to filter out those interactions which have a biological process score less than 0.5. There were 11765 interactions left in our application.

The prediction model constructed on the CombinedYeast datasets using all the true yeast complexes from MIPS and SGD as the positive training data was also applied to a large human signaling network (HSN) to detect new human complexes. HSN (Version 6) contains 6305 proteins and 62937 relations including activation, inhibition and physical interactions (http://www.cancer-systemsbiology.org/dataandsoftware.htm). Physical relations represent complexes that play an important role in cell signaling. To the best of our knowledge, this network has been considered as the largest manually curated human signaling network according to the literature^42^,^43^,^44^,^45^,^46^.

We also combined the two human PPI networks HSN and HPRD into one large human protein relation network (HSN + HPRD). The merged network contains 92252 distinct interactions after removing those duplicates. Then, we predicted human complexes from this network through the model constructed on CombinedYeast using complexes from MIPS and SGD.

To assess the quality of the predicted human complexes, they are compared against the gold standard database CORUM (the comprehensive resource of mammalian protein complexes)^47^ which stores 1843 human protein complexes. We also compared the prediction performance of ClusterEPs with CluseterONE which had the second best performance for predicting yeast complexes shown in the above experiments. As can be seen in Table 6, ClusterEPs achieved a higher Precision, Recall, F1 and MMR scores than ClusterONE. This superior performance is mainly attributed to the knowledge and principles learned by the prediction model from the well explored yeast PPI and complex data.

HSN + HPRD is a very large network, it may contain some noise interactions. Similarly as preprocessing HPRD, we also used TCSS to filter out those interactions having a biological process score less than 0.5. The processed PPI network is named HSNHPRD_F which contains 38091 interactions. We ran our algorithm and ClusterONE on HSNHPRD_F to identify new human complexes. Experimental results (Table 6) show that both our method and ClusterONE achieved a better performance than those on the original HSN + HPRD network. Again, our method achieved a better performance than ClusterONE.

### Case studies

This section first presents detailed case studies on three non-overlapping yeast complexes (i.e. complexes that are separated one another in the yeast PPI networks), a pair of overlapping yeast complexes, and a human protein complex. The three non-overlapping complexes are: the RNA polymerase I complex (large size), RecQ helicase-Topo III complex (small size), and the DASH complex. The two overlapping complexes are the pair of the RSC and the SWI/SNF complexes. The DASH complex and the two overlapping complexes have been previously used as case study examples by ClusterONE^16^ and other methods for performance evaluation. The example of human complex is the ubiquitin E3 ligase complex which was detected by our prediction model constructed on the yeast interaction data.

**Figure 2:**
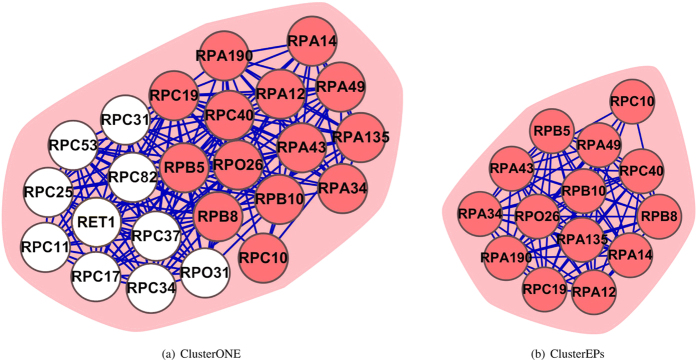
The RNA polymerase I complex as predicted by ClusterONE and ClusterEPs. The red nodes represent proteins that belong to the true complex, while the white color nodes represent proteins that do not belong to the true complex. The shaded area indicates the whole predicted subgraph.

The RNA polymerase I complex is a transcription complex (MIPS identifier 510.10), comprising 14 proteins. The Collins PPI network contains the subgraph of this complex. Figure 2 shows the predicted subgraph of this complex by ClusterEPs in comparison with ClusterONE. Our ClusterEPs could completely identify all the proteins in this complex with 100% precision, while the other seven methods failed to do so. The RNA polymerase I complex subgraph is a subgraph with a density of 0.89. This subgraph also has a higher cohesiveness than sparse subgraphs. However, this subgraph is not a well-separated subgraph, as it is connected to 109 external proteins outside the complex (see Supplementary Figure 4). This means that it has a higher boundary weight. Due to these facts, ClusterONE was not able to exactly recover this complex—it added 10 nearby proteins, which almost doubled the true size of this complex. As a comparison, MCL and CFinder added other proteins into the RNA polymerase I complex. Interestingly, the wrongly predicted proteins by CFinder not only included those added by MCL and ClusterONE, but also included 34 other unrelated proteins. RNSC and RRW were only able to cluster part of the RNA polymerase I. CMC clustered part of this complex and added an unrelated protein. MCODE clustered 30 proteins 10 of which belong to this complex and 20 of which are irrelevant to the RNA polymerase I complex. More details of the results for this complex can be found in Supplementary Figure 5.

**Figure 3:**
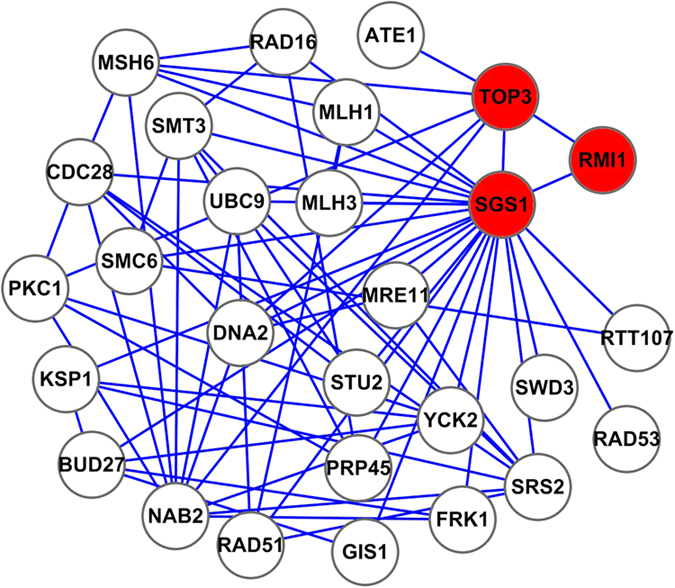
The RecQ helicase-Topo III complex as predicted by ClusterEPs. The red nodes represent proteins that belong to the true complex and the white color nodes represent proteins that do not belong to the true complex.

The RecQ helicase-Topo III complex is a small complex in the SGD database, comprising 3 proteins. Figure 3 shows the neighborhood of this complex in the BioGRID PPI network. Predicting small complexes (consisting of fewer than four proteins) is a challenging research because they are more susceptible to noise (missing or spurious interactions) in a PPI network^48^. The strategy of identifying dense regions (applicable to large complexes) is less effective for the prediction of small complexes^10^. Especially, in the cases where a small complex directly connects to a large number of external proteins, it is even more challenging to detect these small complexes with 100% precision^10^. As our ClusterEPs method not only considers the density of subgraphs, but also makes use of other topological measurements, such as the clustering coefficient that measures the number of triangles that go through a node^49^, the topological coefficient that reflects the number of rectangles that pass through a node^24^,^50^. These measurements can capture and characterize the connectivity of the neighboring subgraphs. Therefore, ClusterEPs was able to completely identify the subgraph of the RecQ helicase-Topo III complex with 100% precision. Other methods (except MCL) could not completely identify this complex.

The DASH complex has been examined as a case study by^16^. The subgraph of this complex was included in the Krogan extended PPI network, and the complex is described by MIPS which contains 9 proteins. Only ClusterONE and ClusterEPs were able to detect this complex completely and correctly. Supplementary Figure 6 presents more details of the result.

The RSC and the SWI/SNF complexes are a pair of overlapping complexes which were also investigated by^16^ as a case study. This pair of overlapping complexes have a match with two overlapping subgraphs in the Collins PPI network. Both ClusterEPs and ClusterONE obtained a prediction result closest to the ground truth. Supplementary Figure 7 presents the detailed prediction result.

**Figure 4:**
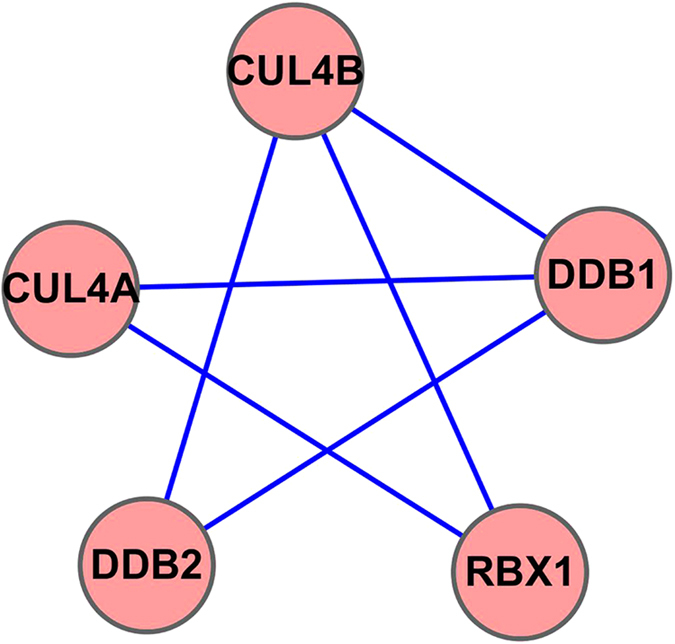
The Ubiquitin E3 ligase complex as predicted by ClusterEPs.

The ubiquitin E3 ligase complex (complexe id:1162) is a human complex recorded at the CORUM database. The topological structure of this complex is like a five-pointed star shape (Fig. 4) in the HPRD PPI network. We constructed the prediction model by training on the yeast complexes data and the six yeast PPI networks. The prediction model was applied to detect human complexes from the HPRD PPI network. The ubiquitin E3 ligase complex was detected when the prediction model was constructed using every individual yeast PPI network. For comparison, we also applied ClusterONE^16^ on the same HPRD PPI network. The result is that an unrelated protein DTL was added into the ubiquitin E3 ligase complex. As only one protein (DDB1) in the whole HPRD PPI network interacts with the protein DTL, the cohesiveness of the subgraph of ubiquitin E3 ligase complex becomes locally optimal after DTL is added to this subgraph as determined by ClusterONE. This is the direct reason why ClusterONE couldn’t detect this complex correctly.

Similar to other supervised learning methods for protein complex prediction, ClusterEPs constructed the negative examples of data by randomly selecting subgraphs. However, this did not seem to have effect on these case studies. We ran ClusterEPs 20 times on two different personal computers, each run ended up with the same prediction results for these case study complexes.

### Predicting currently unknown but biologically interpretable complexes

**Figure 5:**
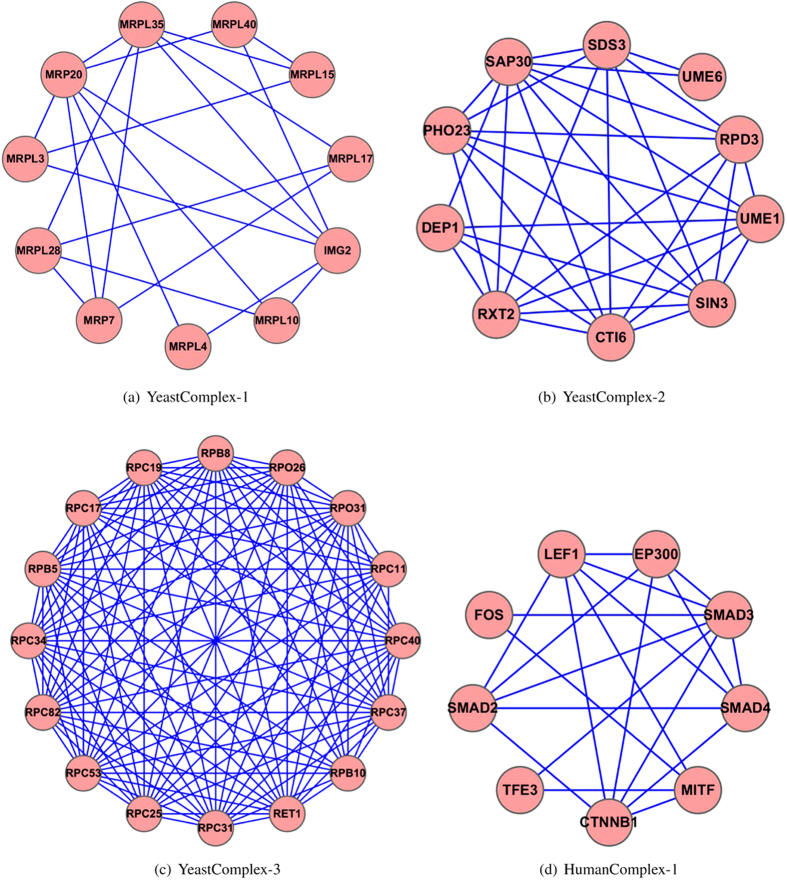
Four examples of complexes identified by ClusterEPs.

Figure 5 presents four examples of identified subgraphs by ClusterEPs. The first complex (YeastComplex-1 in the Krogan core PPI network) is very sparse; while the third one (YeastComplex-3 in the Collins PPI network) is very dense. The second complex YeastComplex-2 is embedded in the Krogan extended PPI network. These three predicted complexes have not been characterized by MIPS or SGD. The fourth example is a subgraph identified from the human PPI network (HPRD) by ClusterEPs when it was trained on the combined yeast PPI network (CombinedYeast). To our best knowledge, this subgraph has not been characterized by any databases including CORUM. Our analysis revealed that none of the four subgraphs contained any EPs favoring the non-complex class, but only EPs favoring the complex class. (Supplementary Tables 28–31 list the features and their values of these subgraphs, and Supplementary Tables 11–16 provide the detailed EPs). That is, these subgraphs match many properties of currently true complexes, but they do not match any property of the random subgraphs. Therefore, these subgraphs are highly likely to be *currently unknown* true complexes.

We conducted gene ontology (GO) enrichment analysis for these predicted complexes by using BINGO^51^. YeastComplex-1 is a sparse subgraph (density 0.4) in the Krogan core PPI network. All the 11 proteins of YeastComplex-1 are enriched in 13 GO terms that are mostly related to translation, macromolecule biosynthetic process, protein metabolic process, gene expression, biosynthetic process, or metabolic process (with a p-value < 8.1*10^−3^). All the 10 genes in YeastComplex-2 are enriched in 56 GO terms that are mostly related to histone deacetylation, protein amino acid deacetylation, positive regulation of transcription, positive regulation of metabolic process, positive regulation of biosynthetic process, negative regulation of cellular process, or post-translation protein modification (with a p-value < 7.891*10^−3^). All the 16 proteins in YeastComplex-3 are enriched in 19 GO terms that are mostly related to transcription from RNA polymerase III promoter, RNA biosynthetic process, RNA metabolic process, or biosynthetic process (with a p-value < 9.076*10^−4^). All the 9 proteins in HumanComplex-1 are enriched in 32 GO terms that are mostly related to positive regulation of transcription, positive regulation of RNA metabolic process, positive regulation of gene expression, positive regulation of metabolic process, positive regulation of biological process, regulation of biosynthetic process, regulation of metabolic process, regulation of cellular process, and biological regulation (with a p-value < 1.489*10^−3^).

These GO analysis results suggest that the proteins in each of these subgraphs are strongly correlated with each other in terms of the enriched GO terms. These subgraphs represent biologically interpretable complexes that are currently not characterized but are worth further experimental investigations. Supplementary Tables 32–35 provide detailed results of these gene ontology enrichment analysis.

Functional information of proteins such as biological processes, molecular functions and cellular components are useful to decide whether two proteins should be classified into or not into the same complex^9^. As proposed by^9^,^22^, proteins similar in specific subsets of these three functional categories should be considerably more preferred than those where proteins are not similar. There are a total of 55 protein pairs in YeastComplex-1. Surprisingly, the cellular component score of every protein pairs in this sugraph achieved the maximum value of 1.0. Biological process scores of 62% (28/45) protein pairs in YeastComplex-2 achieved the maximum value of 1.0. Biological process scores of 87.5% (105/120) protein pairs in YeastComplex-3 achieved the maximum value of 1.0. Molecular function scores of 55.6% (20/36) protein pairs in HumanComplex-1 achieved the maximum value of 1.0, and 13.9% (5/36) protein pairs in HumanComplex-1 had molecular function scores less than 1.0 but greater than 0.9. The functional information scores of the proteins in these identified subgraphs strongly indicate that these subgraphs are worth further experimental investigations for complex verification.

A very recent study^8^ shows that a protein complex is associated with a disease phenotype if one or more of its protein elements are associated with the disease phenotype. With this hypothesis, we can infer that HumanComplex-1 is a disease-protein complex, because SMAD4, MITF, SMAD3, EP300 and CTNNB1 of this subgraph are all known disease genes. For example, Juvenile polyposis syndrome[MIM:174900], Pancreatic cancer (PNCA) [MIM:260350], syndrome phenotype consisting of the coexistence of juvenile polyposis (JIP) and hereditary hemorrhagic telangiectasia (HHT) [MIM:187300], Colorectal cancer (CRC) [MIM:114500], Myhre syndrome (MYHRS) [MIM:139210], and Juvenile polyposis/hereditary hemorrhagic telangiectasia syndrome (JP/HHT) [MIM:175050] are all caused by mutations in SMAD4 [UniProt: Q13485]. Waardenburg syndrome 2A (WS2A) [MIM:193510], Waardenburg syndrome 2, with ocular albinism, autosomal recessive (WS2-OA) [MIM:103470], Tietz syndrome (TIETZS) [MIM:103500], Melanoma, cutaneous malignant 8 (CMM8) [MIM:614456] are all caused by mutations in MITF [UniProt: O75030]. Colorectal cancer (CRC) [MIM:114500] and Loeys-Dietz syndrome 3 (LDS3) [MIM:613795] are both caused by mutations in SMAD3 [UniProt: P84022]. Rubinstein-Taybi syndrome 2 (RSTS2) [MIM:613684] is caused by a mutation in EP300 [UniProt: Q09472]. Pilomatrixoma (PTR) [MIM:132600], Medulloblastoma (MDB) [MIM:155255], Colorectal cancer (CRC) [MIM:114500], Ovarian cancer (OC) [MIM:167000], Mesothelioma, malignant (MESOM) [MIM:156240], and Mental retardation, autosomal dominant 19 (MRD19) [MIM:615075] are all caused by mutations in CTNNB1[UniProt:P35222].

For the other proteins in HumanComplex-1, their functional analysis and results are even more interesting. As discussed by^52^, even if two protein sequences do not share significant sequence identity but share a functional domain, then mutations may disrupt the same process of them and thereby lead to similar phenotypes. SMAD2, SMAD4 and SMAD3 have MH1 and MH2 domains in common [PFAM: PF03165, PF03166]. TFE3 and MITF have DUF3371 and HLH domains in common [PFAM: PF11851, PF00010]. Moreover, SMAD2 and SMAD4 are 77% identical at sequence level, TFE3 and MITF are 77% identical at sequence level. Therefore, we can infer that SMAD2 may have similar disease phenotypes as SMAD4 and SMAD3, and TFE3 may have similar disease phenotypes as MITF.

## Discussion

Due to the fact that gold standard protein complex sets are incomplete^53^, random subgraphs may contain some unknown complexes, although the probability is extremely low. Here unknown complexes mean true complexes but they are not discovered by the research community. ClusterEPs is developed based on the knowledge of true complexes. These unknown complexes that were treated as negative samples in the current work might have an effect on the performance of ClusterEPs. With the increasing availability of true complexes discovered, it is likely to achieve a better performance of ClusterEPs (Supplementary Discussion). In our future work, we will consider exploring positive-unlabeled (PU) learning algorithms^54^,^55^ to achieve a better optimization of the complex search process to further enhance the performance and quality of our method.

There remain significant challenges for the detection of human complexes from human PPI networks for the following reasons and facts:

1. Human PPIs are more noisy and less complete than yeast PPIs. The most recent version of HPRD (Release 9) has collected 39240 interactions, containing a high proportion of human PPIs that are present in other databases^41^, and it includes all human disease-associated genes listed in OMIM. HPRD contains 84.7% (1562 of 1843) human complexes in the^47^(CORUM) database, and 3.7% (23 of 622) human soluble protein complexes (HSPC)^56^. HSPC complexes were not used as a test set to evaluate the predicted complexes in our experiments, because a large percentage of HSPC complexes (96.3%) were not included in human PPIs. However, the yeast PPIs curated by BioGRID contain 96.4% (188 of 195) yeast complexes in MIPS, and 99.7% (322 of 323) yeast complexes in SGD. These facts highlight that human PPIs are actually more noisy and less complete than yeast PPIs.
2. Human PPIs contain a larger number of small complexes compared with yeast PPIs. The CORUM database contains 72.3% (1333 of 1843) of small human complexes (less than five proteins each). HSPC contains 64.3% (400 of 622) of small human complexes. However, MIPS contains 34.9% (68 of 195) of yeast complexes, while SGD contains 61.6% (199 of 323) of yeast complexes. As aforementioned, small complexes are more susceptible to noise (missing or spurious interactions), and accordingly it is a big challenge to detect small complexes accurately.
3. Some human complexes are very big. The maximum size (number of proteins in a complex) of human complexes in CORUM is 142, and the number is 105 in HSPC. However, the maximum size of yeast complexes in MIPS is 95, and it is 55 in SGD. These complexes consist of some small complexes, so it is difficult to recover these complexes.
4. There are many paralogous proteins that have similar and overlapping functions with human PPIs. For example, the proteins AKT1, AKT2 and AKT3 are present in human PPIs, but yeast PPIs have only one AKT. These paralogous proteins have similar and overlapping functions and are involved in multiple complexes. Thus, it remains a big challenge using the sequence or functional information only to identify which protein belongs to which complex.
5. There are many proteins that match many UniProtKB human IDs in human PPIs. For example, the UniProtKB human IDs of the protein HLA-A include P30443, P01892, P04439, P13746, P30447, P05534, P18462, P30450, P30512 and P16188. However, yeast proteins have unique SGD gene IDs. Thus, it remains another difficult challenge as to how to filter out false interactions in order to improve the quality of predicted human PPIs.

ClusterONE^16^ is an unsupervised clustering methods with a better performance than other unsupervised clustering methods. Our ClusterEPs is a supervised learning methods developed based on the knowledge of true protein complexes. When using true yeast complexes to construct the prediction model to detect human complexes from human PPI networks, ClusterEPs achieved a better performance than ClusterONE. This indicates that human complexes have similar topologies as yeast complexes. Thus, this represents an alternative strategy to research human complexes using yeast complexes topologies.

## Methods

A PPI network 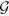 is modeled by an undirected graph 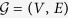, where *V* denotes the set of proteins and *E* denotes the set of protein interactions. The degree of *v* ∈ *V* is defined as the number of its direct neighbors. Let *C* = (*V*_*c*_, *E*_*c*_) be a subgraph in the PPI network. The average degree *avedeg*(*C*) of *C* is given by


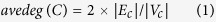


where |*E*_*c*_| denotes the number of edges in *E*_*c*_ and |*V*_*c*_| denotes the number of vertices in *C*. The density *den*(*C*) of *C* is given by


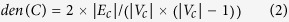


The neighboring nodes of *C* in 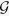 is defined as


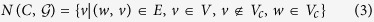


The three major steps of ClusterEPs: Step 1 uses a feature vector to represent the positive class of data (the true complexes) and the negative class of data (the random subgraphs); Step 2 discovers EPs that can distinguish the properties between the complex class and the random subgraph class; Step 3 uses an EP-based scoring method to discover currently unknown protein complexes or re-discover currently known complexes.

### Feature vector representation for true complexes and random PPI subgraphs

Given a subgraph *C* of a PPI graph 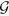, we convert some properties of *C* into a feature vector. This feature vector consists of 22 features. Feature-1 is named *Node Size* (the number of nodes in the subgraph *C*); Feature-2 is named *Graph Density* (the density of the subgraph *C*). The remaining 20 features are organised into six groups: Degree Statistics, Degree Correlation Statistics, Clustering Coefficient Statistics, Topological Coefficients, First Eigenvalues, and Protein Weight/Size Statistics. (See Supplementary Table 1 for details).

For each protein complex subgraph of 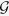, mapped from the gold standard MIPS database or the SGD database, we calculate the values of the 22 features for this subgraph, and represent them as a vector (i.e. a set of feature values). This vector is an instance in the positive class.

To construct a negative dataset, we generate complex-unlikely subgraphs by randomly selecting nodes in the input PPI network, with the number of generated random subgraphs 20 times the true complexes. The size distribution of true complexes in MIPS, SGD or TAP06 is distributed as a power law. In line with other supervised learning methods, we generated non-complexes subgraphs following the same power law distribution. More details can be found in the size distribution of the non-complexes subgraphs of the Supplementary file. We then calculate all the feature values for each random subgraph to be an instance in the negative class.

We use *D*_*p*_ and *D*_*n*_ to denote the positive and negative class, respectively, and use *D* to denote 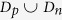.

### Discovery of emerging patterns

For the dataset *D* derived from the input PPI network, a discretization method in the WEKA package^57^ is used to uniformly discretize each feature into 10 equal width bins. A bin of each feature is called an item of the feature. A set of items from different features is called an itemset (pattern).

This work focuses on a special type of EPs called noise-tolerant EPs.

**Definition 1**^58^ *Give two support thresholds δ*_1_ > *0 and δ*_2_ > 0, *δ*_2_ >> *δ*_1_, *a noise-tolerant emerging pattern (NEP) from D*_*1*_ *to D*_*2*_ *is an itemset X that satisfies the following conditions*:

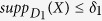
 *and* 
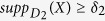
;
2. *any proper subset of X does not satisfy condition 1.*

*where* 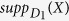 *represents the occurrence frequency of X in dataset D*_1_. 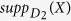 *is similarly defined*.

NEPs have strong predictive power and noise tolerance. An NEP from *D*_1_ to *D*_2_ is simply called an NEP of *D*_2_. The contrast pattern tree algorithm^59^ is modified to discover NEPs from the discretized *D*.

### Searching for new protein complexes

For a PPI network 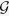, we use *EP*(*D*) to denote the set of NEPs discovered from *D* (namely, NEPs of *D*_*p*_ and NEPs of *D*_*n*_). Assuming *G* is a subgraph of 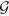, we use *Ins*(*G*) to denote the feature vector comprising of the 22 discretized feature values of *G*. An aggregate score for *G* in terms of *D*_*p*_ is defined as


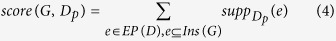


where *e* ⊆ *Ins*(*G*) means *e* is contained by *Ins*(*G*). Similarly, an aggregate score for *G* in terms of *D*_*n*_ is defined as


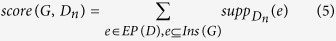


To label the subgraph *G, score*(*G, D*_*p*_) favors a positive label. The larger *score*(*G, D*_*p*_) is, the more likely *G* belongs to the class of true complexes. On the other hand, *score*(*G, D*_*n*_) favors a negative label for *G*. The number of NEPs for the two classes can greatly affect the final decision. If *D*_*p*_ has many more NEPs than *D*_*n*_, *score*(*G, D*_*p*_) would be often larger than *score*(*G, D*_*n*_), even when *G* actually belongs to the negative class. Therefore, we normalize the two scores for making a fair decision.

For each instance of *D*_*p*_, we calculate the score using Equation 4. Then we choose the median value of the |*D*_*p*_| number of scores as a base score for *D*_*p*_, denoted by *base*_*score*(*D*_*p*_). The normalized score for *G* in terms of *D*_*p*_ is given by


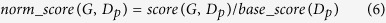


The base score for *D*_*n*_, *base*_*score*(*G, D*_*n*_), can be similarly defined, which is given by


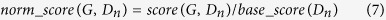


A clustering score for *G* is defined as


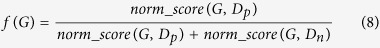


*f*(*G*) > 1/2 means *norm*_*score*(*G, D*_*p*_) > *norm*_*score*(*G, D*_*n*_); it implies that *G* matches more properties of currently known complexes. *f*(*G*) = 1 means *norm*_*score*(*G, D*_*n*_) = 0; it implies that *G* does not match any property of the random subgraphs. Thus, a larger *f*(*G*) suggests that *G* is more likely to be a protein complex.

We search for those subgraphs *G* in 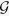 that have a high *f*(*G*). As the number of possible subgraphs in 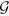 is exponential, we use a heuristic method to identify good candidates for those subgraphs that are likely to be protein complexes.

Using this heuristic method, we first select a seed protein as the starting cluster, and then add proteins one-by-one from its neighbor nodes to grow the cluster. More specifically, we select the protein with the highest degree as the first seed protein from the input PPI network 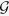. When the cluster grows into a protein complex, the growth process finishes for the current seed protein. We then select the next seed protein with the highest degree from the set of proteins that have not occurred in any of the protein complexes found so far. The search algorithm terminates when each protein in 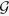 has occurred at least once in the protein complexes found.

The steps of the heuristic search are detailed below. Suppose the search starts from *v*_0_. Step 1: Denote the initial cluster as *C* = {*v*_0_}; Step 2: Compute *f*(*C*) and *avedeg*(*C*). Let *V* = *C*; Step 3: For each vertex *v* in its neighborhood 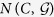 (i.e., Eqn (3)), let *edgeNum*_(*v*,*C*)_ denote the number of edges that connect *v* to the nodes of *C*. We select the vertex *v* with the maximum value of *edgeNum*_(*v*,*C*)_, and then let 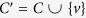. If *f*(*C*′) ≥ *f*(*C*) and *avedeg*(*C*′) > *avedeg*(*C*) then let *C* = *C*′. Step 4: If *C* ≠ *V*, go back to Step 2. Otherwise, if *f*(*C*) > 1/2, then a subgraph *C* is constructed which is predicted to be a true complex.

We note that the search process uses *v* to expand the current subgraph *C* under the two strong conditions: 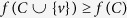 and 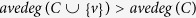. The first condition ensures that a subgraph should grow into a protein complex (i.e., *f*(*G*) > 0.5), while the second condition guarantees that a protein complex should have the highest average degree compared with all its ancestor subgraphs.

As proposed by^16^, we also merge two subgraphs found above if they are highly overlapping. Suppose *C*_1_ and *C*_2_ are two clusters, the overlapping score *ω*^11^ between *C*_1_ and *C*_2_ is defined as


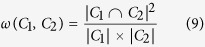


when *ω*(*C*_1_, *C*_2_) meets a certain threshold, then they will be merged as a subgraph 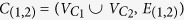, where 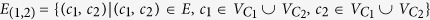.

### Benchmark datasets and performance evaluation measures

The DIP database^33^ has been widely used by supervised learning methods of protein complex detection. The most recent version of DIP PPI networks (1 July 2015) contains 22895 interactions. After removing self-interactions, the resulting DIP network contains 22277 interactions and 4931 proteins. MIPS^23^ and TAP06^36^ are usually used by the supervised learning methods as the positive training set and test set. MIPS contains 103 complexes and TAP06 contains 491 complexes, respectively. Actually, TAP06 is the core set of protein complexes from a TAP-MS experiment.

We used five large-scale yeast PPI datasets to compare the performance of our methods with unsupervised clustering methods. These five PPI datasets are: the Gavin dataset^36^, the Krogan core dataset^38^, the Krogan extended dataset^38^, the Collins dataset^28^, and BioGRID^39^. MIPS^23^ and SGD^34^, as two reference complex sets, were used to compare the quality of predicted complexes. These five PPI datasets and two reference complex sets have been used by ClusterONE^16^ to assess the performance of eight unsupervised clustering methods. Refer to Supplementary Tables 2–3 for details of these datasets.

We used the above six yeast PPI datasets as the training data to detect human complexes from human PPI networks. The Human Protein Reference Database (HPRD)^40^ has been widely used by the research community. HPRD (Release 9) has collected 39240 interactions, containing a high proportion of human PPIs that are present in other literature databases^41^. We removed self-interactions from HPRD, and the resulting HPRD network contains 37080 interactions and 9465 proteins. We used 1843 human protein complexes from the database of the comprehensive resource of mammalian protein complexes^47^(CORUM) as gold standards.

Three independent quality measures are used to assess the performance of supervised learning methods, i.e. Precision, Recall and F1-measure. Let *S* denote the set of standard protein complexes (true complexes), and *P* denote the set of predicted protein complexes by a method. The set of true complexes detected by predicted complexes is defined as *S*_*t*_ = {*s* | *ω*(*s, p*) ≥ 0.25, *s* ∈ *S, p* ∈ *P*}, and the set of predicted complexes matching true complexes is defined as *S*_*p*_ = {*p* | *ω*(*s, p*) ≥ 0.25, *s* ∈ *S, p* ∈ *P*}. Precision and Recall are then given by |*S*_*p*_|/|*P*| and |*S*_*t*_|/|*S*|, respectively, where |*S*| and |*P*| denotes the numbers of standard complexes and predicted complexes, respectively. The F1-measure is defined as 2*|*S*_*p*_|/|*S*_*t*_|.

Recall measures the fraction of true complexes matched by at least one predicted complex. It is also denoted as the fraction of matched complexes in the literature^16^. In this work, we use “Frac” and “Recall” interchangeably to represent the fraction of matched complexes.

A predicted complex and a gold standard one are often matched in part. A gold standard complex may match with more than one predicted complexes, and vice versa. These facts make the comparison difficult^16^. To address this problem, Nepusz *et al.* proposed a measure based on the maximum matching ratio (MMR), which is based on the maximal one-to-one mapping between predicted and the standard complexes^16^. MMR builds on a bipartite graph, in which each node represents a standard protein complex or a predicted protein complex. A maximum matching ratio is calculated as follows:

1. For each standard protein complex *s* ∈ *S* and predicted protein complex candidate *p* ∈ *P*, if *ω*(*s, p*) > 0, then *s* and *p* are connected by an edge with the weight equal to *ω*(*s, p*).
2. A subset of edges *subEdges* is chosen from the bipartite graph such that these edges satisfy the two following conditions:
  1. For each *s* ∈ *S* and *p* ∈ *P, s* and *p* are incident on at most one of the selected edges.
  2. The sum of the weights of the selected edges is maximal.

Then, the MMR between *S* and *P* is the sum of the weights of the selected edges in *subEdges*, divided by |*S*|.

Another independent measure is the geometric accuracy (*Acc*)^35^. *Acc* is the geometrical mean of the clustering-wise sensitivity *Sen*(*S, P*) and the clustering-wise positive predictive value *PPV*(*S, P*). Suppose *S* = {*s*_1_, *s*_2_, ···, *s*_*i*_, ···, *s*_*n*_} contains *n* standard protein complexes, and *P* = {*p*_1_, *p*_2_, ···, *p*_*j*_, ···, *p*_*m*_} contains *m* predicted protein complexes, where *s*_*i*_ and *p*_*j*_ respectively denotes the *i*^*th*^ standard protein complex and the *j*^*th*^ predicted protein complex, respectively. Let ***T*** be a matrix with *n* rows and *m* columns, row *i* corresponds to *s*_*i*_ and column *j* corresponds to *p*_*j*_. *T*_*ij*_ indicates the number of proteins found both in *s*_*i*_ and *p*_*j*_. *Sen*(*S, P*) is defined as


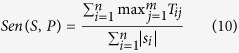


where, |*s*_*i*_| is the number of proteins in the complex *s*_*i*_. *PPV*(*S, P*) is given by


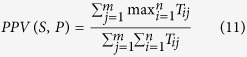


Then,


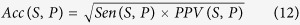


## Additional Information

**How to cite this article**: Liu, Q. *et al.* Using contrast patterns between true complexes and random subgraphs in PPI networks to predict unknown protein complexes. *Sci. Rep.* **6**, 21223; doi: 10.1038/srep21223 (2016).

## Supplementary Material

Supplementary Information

## Figures and Tables

**Figure 1:**
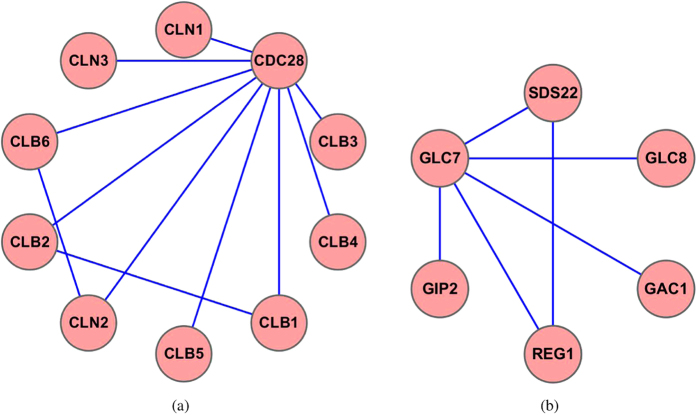
Sparse complexes in the MIPS complex catalogue database.

**Table 1:** Examples of emerging patterns from the Collins PPI network.

Emerging patterns	Frequency (%) in subgraphs representing true complexes	Frequency (%) in random subgraphs
{*meanDegree* > 0.61}	99.4	0
{*meanClusteringCoeff* > 0.3}	88.6	0
{*meanDegreeCorrelation* ≤ 1.9, *maxLength* > 868}	2.4	85

**Table 2:** Performance comparison with SCI-BN and RM.

Train	Test	Method	Precision	Recall	F1
MIPS	TAP06	**ClusterEPs**	0.399	**0.798**	**0.529**
MIPS	TAP06	SCI-BN	0.312	0.489	0.381
MIPS	TAP06	SCI-SVM	0.247	0.377	0.298
MIPS	TAP06	RM	**0.424**	0.433	0.429
TAP06	MIPS	**ClusterEPs**	**0.601**	**0.672**	**0.634**
TAP06	MIPS	SCI-BN	0.219	0.537	0.312
TAP06	MIPS	SCI-SVM	0.176	0.379	0.240
TAP06	MIPS	RM	0.489	0.525	0.506

**Table 3:** Performance comparison with NN.

Train	Test	Method	Precision	Recall	F1
MIPS	MIPS	ClusterEPs	**0.638**	**0.769**	**0.696**
MIPS	MIPS	SCI-BN	0.273	0.473	0.346
MIPS	MIPS	SCI-SVM	0.239	0.412	0.302
MIPS	MIPS	RM	0.419	0.67	0.514
MIPS	MIPS	NN	0.333	0.491	0.397

**Table 4:** Performance comparison of eight algorithms tested on five yeast PPI datasets using MIPS as the test set.

Datasets	Methods	#cluster	Frac	Acc	MMR	Composite score
Gavin	ClusterEPs	240	**0.774**	0.479	**0.431**	**1.684**
	MCL	252	0.681	**0.503**	0.331	1.515
	MCODE	135	0.611	0.447	0.301	1.359
	CMC	339	0.663	0.452	0.347	1.462
	ClusterONE	196	0.708	0.5	0.375	1.583
	RNSC	138	0.611	0.485	0.319	1.415
	RRW	234	0.664	0.446	0.34	1.45
	CFinder	137	0.558	0.487	0.279	1.324
Krogan core	ClusterEPs	364	**0.691**	0.420	**0.357**	**1.468**
	MCL	376	0.6	**0.441**	0.272	1.313
	MCODE	79	0.326	0.334	0.144	0.804
	CMC	156	0.37	0.374	0.172	0.916
	ClusterONE	522	0.674	0.44	0.319	1.433
	RNSC	87	0.422	0.383	0.182	0.987
	RRW	329	0.504	0.361	0.248	1.113
	CFinder	115	0.341	0.369	0.166	0.876
Krogan extended	ClusterEPs	561	**0.598**	0.386	**0.319**	**1.302**
	MCL	483	0.436	0.409	0.193	1.038
	MCODE	64	0.192	0.294	0.097	0.583
	CMC	421	0.365	0.343	0.172	0.88
	ClusterONE	530	0.577	**0.422**	0.284	1.283
	RNSC	93	0.365	0.369	0.159	0.893
	RRW	232	0.468	0.354	0.222	1.044
	CFinder	121	0.218	0.315	0.107	0.64
Collins	ClusterEPs	171	0.705	0.528	**0.421**	1.654
	MCL	183	0.739	0.537	0.397	1.673
	MCODE	112	0.652	0.499	0.351	1.502
	CMC	184	0.591	0.509	0.309	1.409
	ClusterONE	195	**0.782**	**0.555**	0.418	**1.755**
	RNSC	94	0.608	0.499	0.306	1.413
	RRW	190	0.678	0.446	0.378	1.502
	CFinder	114	0.557	0.495	0.312	1.364
BioGRID	ClusterEPs	862	**0.635**	0.366	**0.304**	**1.304**
	MCL	338	0.196	0.348	0.083	0.627
	MCODE	85	0.201	0.325	0.08	0.606
	CMC	N/A	N/A	N/A	N/A	N/A
	ClusterONE	473	0.466	**0.44**	0.195	1.101
	RNSC	209	0.481	0.43	0.212	1.123
	RRW	253	0.402	0.348	0.176	0.926
	CFinder	N/A	N/A	N/A	N/A	N/A

**Table 5:** Performance comparison of eight algorithms tested on five yeast PPI datasets using SGD as the test set.

Datasets	Methods	#cluster	Frac	Acc	MMR	Composite score
Gavin	ClusterEPs	244	**0.825**	0.657	**0.526**	**2.008**
	MCL	253	0.75	0.689	0.438	1.877
	MCODE	135	0.602	0.628	0.38	1.61
	CMC	339	0.726	0.612	0.466	1.804
	ClusterONE	196	0.789	**0.706**	0.476	1.971
	RNSC	143	0.648	0.684	0.398	1.73
	RRW	237	0.758	0.667	0.471	1.896
	CFinder	137	0.609	0.668	0.369	1.646
Krogan core	ClusterEPs	292	0.642	0.584	**0.431**	1.656
	MCL	367	0.636	0.637	0.354	1.627
	MCODE	79	0.394	0.477	0.218	1.089
	CMC	156	0.394	0.516	0.232	1.142
	ClusterONE	522	**0.667**	**0.663**	0.418	**1.748**
	RNSC	88	0.43	0.529	0.23	1.189
	RRW	264	0.606	0.561	0.361	1.528
	CFinder	115	0.418	0.494	0.243	1.155
Krogan extended	ClusterEPs	522	**0.669**	0.550	**0.414**	**1.633**
	MCL	517	0.492	0.594	0.253	1.339
	MCODE	64	0.278	0.422	0.147	0.847
	CMC	351	0.401	0.513	0.232	1.146
	ClusterONE	530	0.594	**0.628**	0.364	1.586
	RNSC	97	0.406	0.527	0.225	1.158
	RRW	232	0.54	0.529	0.311	1.38
	CFinder	88	0.262	0.47	0.155	0.887
Collins	ClusterEPs	385	0.805	0.646	**0.536**	1.987
	MCL	181	**0.836**	0.723	0.518	2.077
	MCODE	112	0.672	0.66	0.439	1.771
	CMC	184	0.589	0.626	0.37	1.585
	ClusterONE	195	0.828	**0.731**	0.532	**2.091**
	RNSC	95	0.627	0.661	0.377	1.665
	RRW	190	0.754	0.656	0.494	1.904
	CFinder	114	0.605	0.648	0.412	1.665
BioGRID	ClusterEPs	780	**0.668**	0.539	**0.397**	**1.604**
	MCL	335	0.3	0.46	0.144	0.904
	MCODE	85	0.21	0.424	0.094	0.728
	CMC	N/A	N/A	N/A	N/A	
	ClusterONE	481	0.562	**0.628**	0.277	1.467
	RNSC	220	0.527	0.616	0.287	1.43
	RRW	270	0.485	0.534	0.263	1.282
	CFinder	N/A	N/A	N/A	N/A	N/A

**Table 6:** Performance of ClusterEPs in the detection of human complexes through the model constructed on the yeast PPI and complexes data.

Method	Trained yeast PPI	Trained yeast complexes	Human PPI	Precision	Recall	F1	Acc	MMR
ClusterEPs	Gavin	MIPS + SGD	HPRD	0.211	0.603	0.313	0.243	0.206
ClusterEPs	Krogan core	MIPS + SGD	HPRD	0.232	0.551	0.326	0.226	0.195
ClusterEPs	krogan extended	MIPS + SGD	HPRD	0.217	0.586	0.317	0.238	0.207
ClusterEPs	Collins	MIPS + SGD	HPRD	0.191	0.612	0.292	0.247	0.201
ClusterEPs	BioGRID	MIPS + SGD	HPRD	0.197	0.580	0.294	0.239	0.198
ClusterEPs	DIP	MIPS + SGD	HPRD	0.190	0.574	0.286	0.245	0.199
ClusterEPs	CombinedYeast	MIPS + SGD	HPRD	0.208	0.482	0.290	0.213	0.173
ClusterONE			HPRD	0.169	0.376	0.233	0.317	0.114
ClusterEPs	CombinedYeast	MIPS + SGD	HSN	0.121	0.429	0.189	0.248	0.139
ClusterONE			HSN	0.057	0.128	0.079	0.308	0.035
ClusterEPs	CombinedYeast	MIPS + SGD	HSN + HPRD	0.115	0.448	0.183	0.217	0.170
ClusterONE			HSN + HPRD	0.062	0.164	0.090	0.312	0.057
ClusterEPs	CombinedYeast	MIPS + SGD	HSNHPRD_F	0.196	0.504	0.282	0.237	0.171
ClusterONE			HSNHPRD_F	0.125	0.242	0.165	0.332	0.074

## References

[b1] Fiannaca A., La Rosa M., Urso A., Rizzo R. & Gaglio S. A knowledge-based decision support system in bioinformatics: an application to protein complex extraction. BMC Bioinformatics 14, S5 (2013). 23368995 10.1186/1471-2105-14-S1-S5 PMC3548703

[b2] Schwikowski B., Uetz P. & Fields S. A network of protein-protein interactions in yeast. Nat. Biotechnol. 18, 1257–1261 (2000). 11101803 10.1038/82360

[b3] Lage K. *et al.* A human phenome-interactome network of protein complexes implicated in genetic disorders. Nat. Biotechnol. 25, 309–316 (2007). 17344885 10.1038/nbt1295

[b4] Yang P., Li X., Wu M., Kwoh C.-K. & Ng S.-K. Inferring gene-phenotype associations via global protein complex network propagation. PLoS ONE 6, e21502 (2011). 21799737 10.1371/journal.pone.0021502 PMC3143124

[b5] Fraser H. & Plotkin J. Using protein complexes to predict phenotypic effects of gene mutation. Genome Biol. 8, R252 (2007). 18042286 10.1186/gb-2007-8-11-r252 PMC2258176

[b6] Liang Y. *et al.* Inferring drug-disease associations based on known protein complexes. BMC Med Genomics 8, S2 (2015). 10.1186/1755-8794-8-S2-S2 PMC4460611 26044949

[b7] Altaf-Ul-Amin M., Shinbo Y., Mihara K., Kurokawa K. & Kanaya S. Development and implementation of an algorithm for detection of protein complexes in large interaction networks. BMC Bioinformatics 7, 207 (2006). 16613608 10.1186/1471-2105-7-207 PMC1473204

[b8] Le D. H. A novel method for identifying disease associated protein complexes based on functional similarity protein complex networks. Algorithm Mol Biol 10, 1–12 (2015). 10.1186/s13015-015-0044-6 PMC4427953 25969691

[b9] Hu L. & Chan K. C. A density-based clustering approach for identifying overlapping protein complexes with functional preferences. Bmc Bioinformatics 16, 1–16 (2015). 26013799 10.1186/s12859-015-0583-3 PMC4445992

[b10] Srihari S., Yong C. H., Patil A. & Wong L. Methods for protein complex prediction and their contributions towards understanding the organisation, function and dynamics of complexes. Febs Letters 589, 2590–2602 (2015). 25913176 10.1016/j.febslet.2015.04.026

[b11] Bader G. D. & Hogue C. W. An automated method for finding molecular complexes in large protein interaction networks. BMC Bioinformatics 4, 2 (2003). 12525261 10.1186/1471-2105-4-2 PMC149346

[b12] Enright A. J., Van Dongen S. & Ouzounis C. A. An efficient algorithm for large-scale detection of protein families. Nucleic Acids Res. 30, 1575–1584 (2002). 11917018 10.1093/nar/30.7.1575 PMC101833

[b13] Liu G., Wong L. & Chua H. N. Complex discovery from weighted ppi networks. Bioinformatics 25, 1891–1897 (2009). 19435747 10.1093/bioinformatics/btp311

[b14] Palla G., Derenyi I., Farkas I. & Vicsek T. Uncovering the overlapping community structure of complex networks in nature and society. Nature 435, 814–818 (2005). 15944704 10.1038/nature03607

[b15] Adamcsek B., Palla G., Farkas I. J., Derényi I. & Vicsek T. Cfinder: locating cliques and overlapping modules in biological networks. Bioinformatics 22, 1021–1023 (2006). 16473872 10.1093/bioinformatics/btl039

[b16] Nepusz T., Yu H. & Paccanaro A. Detecting overlapping protein complexes in protein-protein interaction networks. Nat. Methods 9, 471–472 (2012). 22426491 10.1038/nmeth.1938 PMC3543700

[b17] Macropol K., Can T. & Singh A. Rrw: repeated random walks on genome-scale protein networks for local cluster discovery. BMC Bioinformatics 10, 283 (2009). 19740439 10.1186/1471-2105-10-283 PMC2748087

[b18] Yong C. H., Liu G., Chua H. N. & Wong L. Supervised maximum-likelihood weighting of composite protein networks for complex prediction. BMC Syst Biol 6, 1–21 (2012). 23281936 10.1186/1752-0509-6-S2-S13 PMC3521185

[b19] Min W., Li X., Kwoh C. K. & Ng S. K. A core-attachment based method to detect protein complexes in ppi networks. Bmc Bioinformatics 10, 1–16 (2009). 19486541 10.1186/1471-2105-10-169 PMC2701950

[b20] Ma X. & Gao L. Predicting protein complexes in protein interaction networks using a core-attachment algorithm based on graph communicability. Inform Sciences 189, 233–254 (2012).

[b21] King A. D., Przulj N. & Jurisica I. Protein complex prediction via cost-based clustering. Bioinformatics 20, 3013–3020 (2004). 15180928 10.1093/bioinformatics/bth351

[b22] Hu A. L. & Chan K. C. C. Utilizing both topological and attribute information for protein complex identification in ppi networks. IEEE ACM T Comput Bi 10, 780–792 (2013). 10.1109/TCBB.2013.37 24091410

[b23] Mewes H. W. *et al.* Mips: analysis and annotation of proteins from whole genomes. Nucleic Acids Res. 32, D41–D44 (2004). 14681354 10.1093/nar/gkh092 PMC308826

[b24] Qi Y., Balem F., Faloutsos C., Klein-Seetharaman J. & Bar-Joseph Z. Protein complex identification by supervised graph local clustering. Bioinformatics 24, i250–i268 (2008). 18586722 10.1093/bioinformatics/btn164 PMC2718642

[b25] Feng Y. Y. *et al.* Predicting protein complex in protein interaction network - a supervised learning based method. BMC Syst Biol. 8, S4 (2014). 10.1186/1752-0509-8-S3-S4 PMC4243764 25349902

[b26] Lei S., Lei X. & Zhang A. Protein complex detection with semi-supervised learning in protein interaction networks. Protemo Sci. 9, 295–297 (2011). 10.1186/1477-5956-9-S1-S5 PMC3289084 22165896

[b27] Dong G. & Li J. Efficient mining of emerging patterns: discovering trends and differences. In *Proceedings of the fifth ACM SIGKDD international conference on Knowledge discovery and data mining,* KDD’ **99**, 43–52 (ACM, New York, NY, USA, 1999).

[b28] Collins S. R. *et al.* Toward a comprehensive atlas of the physical interactome of saccharomyces cerevisiae. Mol. Cell Proteomics 6, 439–450 (2007). 17200106 10.1074/mcp.M600381-MCP200

[b29] Li J. & Wong L. Identifying good diagnostic gene groups from gene expression profiles using the concept of emerging patterns. Bioinformatics 18, 725–734 (2002). 12050069 10.1093/bioinformatics/18.5.725

[b30] Li J., Liu H., Ng S.-K. & Wong L. Discovery of significant rules for classifying cancer diagnosis data. Bioinformatics 19, ii93–ii102 (2003). 14534178 10.1093/bioinformatics/btg1066

[b31] Li J., Liu H., Downing J. R., Yeoh A. E.-J. & Wong L. Simple rules underlying gene expression profiles of more than six subtypes of acute lymphoblastic leukemia (all) patients. Bioinformatics 19, 71–78 (2003). 12499295 10.1093/bioinformatics/19.1.71

[b32] Boulesteix A.-L., Tutz G. & Strimmer K. A cart-based approach to discover emerging patterns in microarray data. Bioinformatics 19, 2465–2472 (2003). 14668233 10.1093/bioinformatics/btg361

[b33] Xenarios I. *et al.* Dip, the database of interacting proteins: a research tool for studying cellular networks of protein interactions. Nucleic Acids Res. 30, 303–305 (2002). 11752321 10.1093/nar/30.1.303 PMC99070

[b34] Hong E. L. *et al.* Gene ontology annotations at sgd: new data sources and annotation methods. Nucleic Acids Res. 36, D577–D581 (2008). 17982175 10.1093/nar/gkm909 PMC2238894

[b35] Brohée S. & van Helden J. Evaluation of clustering algorithms for protein-protein interaction networks. BMC Bioinformatics 7, 488 (2006). 17087821 10.1186/1471-2105-7-488 PMC1637120

[b36] Gavin A.-C. *et al.* Proteome survey reveals modularity of the yeast cell machinery. Nature 440, 631–636 (2006). 16429126 10.1038/nature04532

[b37] Jain S. & Bader G. D. An improved method for scoring protein-protein interactions using semantic similarity within the gene ontology. Bmc Bioinformatics 11, 1–14 (2010). 21078182 10.1186/1471-2105-11-562 PMC2998529

[b38] Krogan N. J. *et al.* Global landscape of protein complexes in the yeast saccharomyces cerevisiae. Nature 440, 637–643 (2006). 16554755 10.1038/nature04670

[b39] Stark C. *et al.* Biogrid: a general repository for interaction datasets. Nucleic Acids Res. 34, D535–D539 (2006). 16381927 10.1093/nar/gkj109 PMC1347471

[b40] Peri S. *et al.* Development of human protein reference database as an initial platform for approaching systems biology in humans. Genome Res. 13, 2363–2371 (2003). 14525934 10.1101/gr.1680803 PMC403728

[b41] Mathivanan S. *et al.* An evaluation of human protein-protein interaction data in the public domain. Bmc Bioinformatics 7, 56–66 (2006). 17254303 10.1186/1471-2105-7-S5-S19 PMC1764475

[b42] Cui Q. *et al.* A map of human cancer signaling. Mol Syst Biol 3, 152 (2007). 18091723 10.1038/msb4100200 PMC2174632

[b43] Awan A. *et al.* Regulatory network motifs and hotspots of cancer genes in a mammalian cellular signalling network. IET Syst Biol 1, 292–297 (2007). 10.1049/iet-syb:20060068 17907678

[b44] Cui Q., Purisima E. & Wang E. Protein evolution on a human signaling network. BMC Syst Biol 3, 21 (2009). 19226461 10.1186/1752-0509-3-21 PMC2649034

[b45] Li L. *et al.* The human phosphotyrosine signaling network: Evolution and hotspots of hijacking in cancer. Genome Res 22, 1222–1230 (2012). 22194470 10.1101/gr.128819.111 PMC3396364

[b46] Zaman N. *et al.* Signaling network assessment of mutations and copy number variations predict breast cancer subtype-specific drug targets. Cell Rep 5, 216–223 (2013). 24075989 10.1016/j.celrep.2013.08.028

[b47] Ruepp A. *et al.* Corum: the comprehensive resource of mammalian protein complexes—2009. Nucleic Acids Res. 38, 497–501 (2010). 10.1093/nar/gkp914 PMC2808912 19884131

[b48] Yong C., Maruyama O. & Wong L. Discovery of small protein complexes from ppi networks with size-specific supervised weighting. BMC Syst Biol 8, S3 (2014). 25559663 10.1186/1752-0509-8-S5-S3 PMC4305982

[b49] Barabasi A.-L. & Oltvai Z. N. Network biology: understanding the cell’s functional organization. Nat Rev Genet. 5, 101–113 (2004). 14735121 10.1038/nrg1272

[b50] Stelzl U. *et al.* A human protein-protein interaction network: a resource for annotating the proteome. Cell 122, 957–968 (2005). 16169070 10.1016/j.cell.2005.08.029

[b51] Maere S., Heymans K. & Kuiper M. Bingo: a cytoscape plugin to assess overrepresentation of gene ontology categories in biological networks. Bioinformatics 21, 3448–3449 (2005). 15972284 10.1093/bioinformatics/bti551

[b52] Vanel M. A., Bruggeman J., Vriend G., Brunner H. G. & Leunissen J. A. A text-mining analysis of the human phenome. Eur J Hum Genet 14, 535–42 (2006). 16493445 10.1038/sj.ejhg.5201585

[b53] Jansen R. & Gerstein M. Analyzing protein function on a genomic scale: the importance of gold-standard positives and negatives for network prediction. Curr Opin Microbiol 7, 535–545 (2004). 15451510 10.1016/j.mib.2004.08.012

[b54] Yang P., Li X.-L., Mei J.-P., Kwoh C.-K. & Ng S.-K. Positive-unlabeled learning for disease gene identification. Bioinformatics 28, 2640–2647 (2012). 22923290 10.1093/bioinformatics/bts504 PMC3467748

[b55] Yang P., Li X., Chua H.-N., Kwoh C.-K. & Ng S.-K. Ensemble positive unlabeled learning for disease gene identification. PLoS ONE 9, e97079 (2014). 24816822 10.1371/journal.pone.0097079 PMC4016241

[b56] Havugimana P. *et al.* A census of human soluble protein complexes. Cell 150, 1068–1081 (2012). 22939629 10.1016/j.cell.2012.08.011 PMC3477804

[b57] Witten I. & Frank E. Data Mining: Practical Machine Learning Tools and Techniques, Morgan Kaufmann Series in Data Management Systems (Morgan Kaufmann, San Francisco, 2005), second edn.

[b58] Fan H. & Ramamohanarao K. Fast discovery and the generalization of strong jumping emerging patterns for building compact and accurate classifiers. IEEE T. Knowl. Data. En . 18, 721–737 (2006).

[b59] Liu Q., Shi P., Hu Z. & Zhang Y. A novel approach of mining strong jumping emerging patterns based on bsc-tree. Int. J. Syst. Sci. 45, 598–615 (2014).

