# Supplementary material for: Using contrast patterns between true complexes and random subgraphs in PPI networks to predict unknown protein complexes
Source: Sci Rep. 2016 Feb 12;6:21223. doi: 10.1038/srep21223 (PMC4751475; doi:10.1038/srep21223)
Supplement: Supplementary Information [file srep21223-s1.pdf]

Using contrast patterns between true complexes and random subgraphs in PPI networks to predict unknown protein complexes

Quanzhong Liu, Jiangning Song, and Jinyan Li

Contents

1 Important properties of subgraphs 2
2 Datasets 2
3 Nondense complexes 3
4 The size distribution of the non-complex subgraphs 4
5 Parameter settings 7
5.1 Support threshold parameters setting for mining NEPs 7
5.2 Parameter settings of ClusterEPs and other tested algorithms 9
6 Mined NEPs in each dataset 9
7 Quality scores of the predicted complexes 13
7.1 Results on five yeast PPI Datasets. 14
7.2 Discussion of the effect of random noise on quality scores 23
8 Case studies 24
9 Prediction of unknown but biologically interpretable complexes 29
9.1 Interpretable complexes 29
9.2 Gene ontology (GO) enrichment analysis 31
References 34

1 Important properties of subgraphs

Supplementary Table 1: Features used for describing subgraph properties. Refer to [1] for more details of these features

Group	Features
Node size	This group includes one feature which represents the number of nodes in the subgraph. The feature is denoted as *nodeSize.*
Graph density	This group includes one feature which represents the density of the subgraph. The feature is denoted as *graphDensity.*
Degree statistics	This group includes four features: mean degree, degree variance, degree median and degree maximum. They are denoted as *meanDegree*, *varDegree*, *medianDegree* and *maxDegree*, respectively.
Degree correlation statistics	This group includes three features: mean degree correlation, degree correlation variance and degree correlation maximum. They are denoted as *meanDegreeCorrelation, varDegreeCorrelation* and *maxDegreeCorrelation,* respectively.
Clustering coefficient statistics	This group includes three features: mean clustering coefficient, clustering coefficient variance and clustering coefficient maximum. They are denoted as *meanClusteringCoeff, varClusteringCoeff* and *maxClusteringCoeff,* respectively.
Topological coefficient	This group includes three features: mean topological coefficient, topological coefficient variance and topological coefficient maximum. They are denoted as *meanTopologicCoeff, varTopologicCoeff* and *maxTopologicCoeff,* respectively.
First Eigenvalues	This group includes three features representing the first three largest singular values of the candidate subgraph's adjacency matrix. They are denoted as *eigenValue*_1, *eigenValue*_2 and *eigenValue*_3, respectively.
Protein weight/size statistics	This group includes four features representing average and maximum protein length and average and maximum protein weight. They are denoted as *aveLength, maxLength, aveWeight* and *maxWeight,* respectively.

2 Datasets

Supplementary Table 2: Properties of the protein-protein interaction datasets

Datasets	Number of proteins	Number of interactions
DIP	4931	22277
Gavin	1855	7669
Krogan core	2708	7123
Krogan extended	3672	14317
Collins	1622	9074
BioGRID	5640	59748
HPRD	9465	37080
HSN	6305	62937

Supplementary Table 3：Properties of gold standard complex datasets

	MIPS	SGD	TAP06	CORUM
Number of proteins	1189	1279	824	2561
Number of complexes	195	323	193	1843
Overlapping complex pairs	353(1.9%)	296(0.6%)	77 (0.4%)	21010 (1.2%)
The max size of complexes	95	55	23	142
Small complexes (consisting of fewer than four proteins)	68 (34.9%)	199 (61.6%)	120 (62.1%)	1333 (72.3%)

3 Nondense complexes

Three complexes are selected from the MIPs complex catalogue database. Supplementary Table 4 shows that the density of these three complex subgraphs in experimental PPI networks. These three complexes do not appear in the Collins and Gavin PPI networks. The asterisk in the table indicates that the corresponding complex does not appear in the corresponding PPI network.

These three complex subgraphs in the corresponding PPI networks are respectively shown in Supplementary Figure 1.

Supplementary Table 4：Sparse complexes selected in the MIPs complex catalogue database

Complex Name	The density in the Krogan core	The density in the Krogan extended	The density in the BioGRID
Cdc28p complexes	*	*	0.24
Threonine phoshpoprotein phosphatase	*	*	0.4
AP-2 complex	0.5	0.5	*

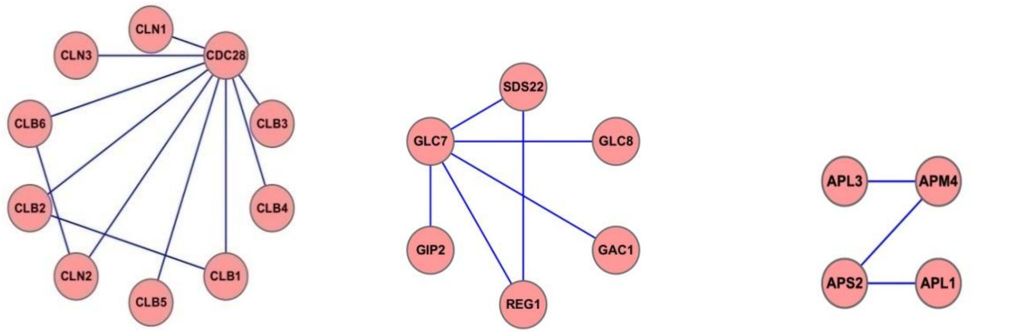

(a) Cdc28p complexes (b) Threonine phoshpoprotein phosphatase (c) AP-2 complex

Supplementary Figure 1: selected yeast MIPS complexes on experimental PPI networks

4 The size distribution of the non-complex subgraphs

Subgraphs generated by randomly selecting nodes in a given PPI network satisfy the following conditions.

(1) Subgraphs are generated by the random generator. But we ensure that each generated subgraph is not a true complex.

(2) The size range of generated subgraphs is the same as that of known complexes appearing in the PPI network. The size distribution of true complexes in MIPS, SGD and TAP06 is distributed as a power law, respectively. Generated subgraphs following the same power law distribution.

(3) Subgraphs generated may be connected; maybe some proteins in a random subgraph are not directly connected to the rest of the subgraph.

A connected subgraph may be an unknown complex, even the subgraph is connected by a linear shape **(**see **Supplementary Figure 1(c))**. If an unknown complex subgraph is selected, then the subgraph will be regarded as a negative example. Ideally, an unknown complex should be regarded as a positive instance. That is to say, an unknown complex subgraph is the noise. But NEPs is a special EPs which can handle with the noise in dataset. Thus, we do not care whether a random subgraph is connected. The size distribution of true complexes and these random subgraphs in experimental PPI networks are shown in **Supplementary Figure 2,** respectively.

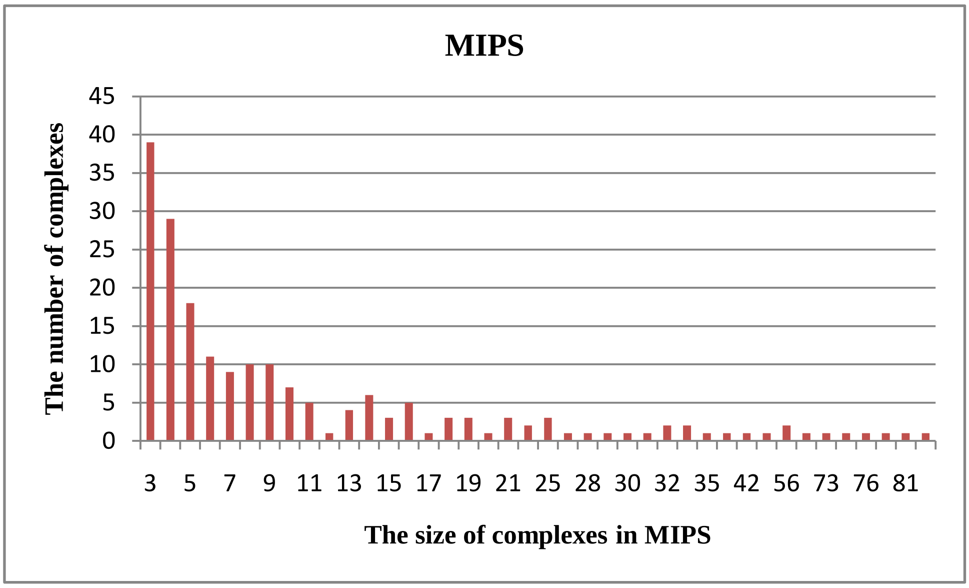

(a) The size distribution of complexes in MIPS

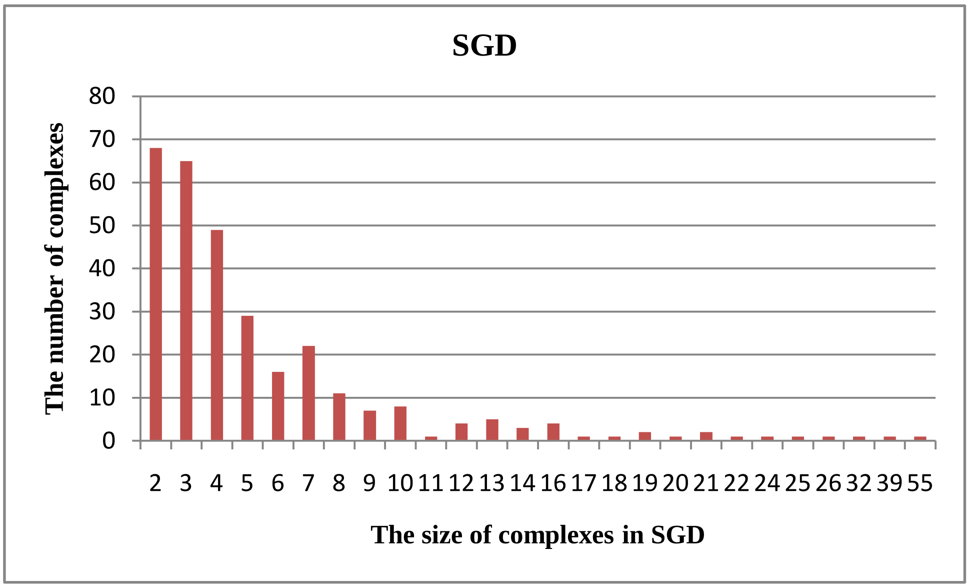

(b) The size distribution of complexes in SGD

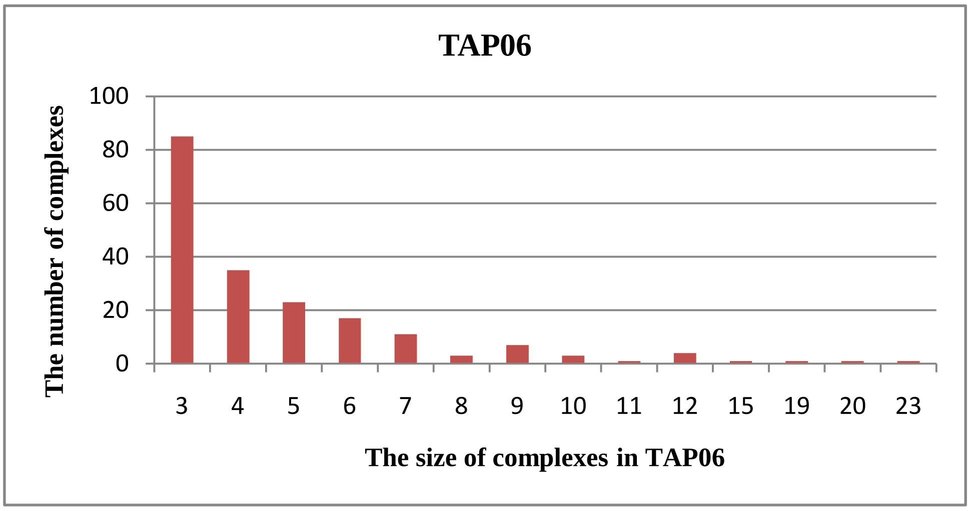

(c) The size distribution of complexes in TAP06

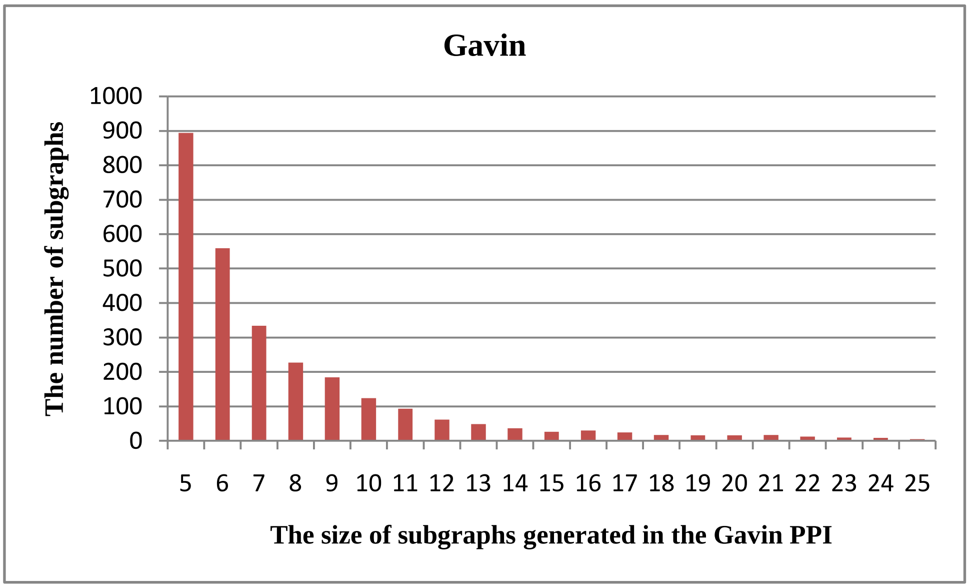

(d) Gavin

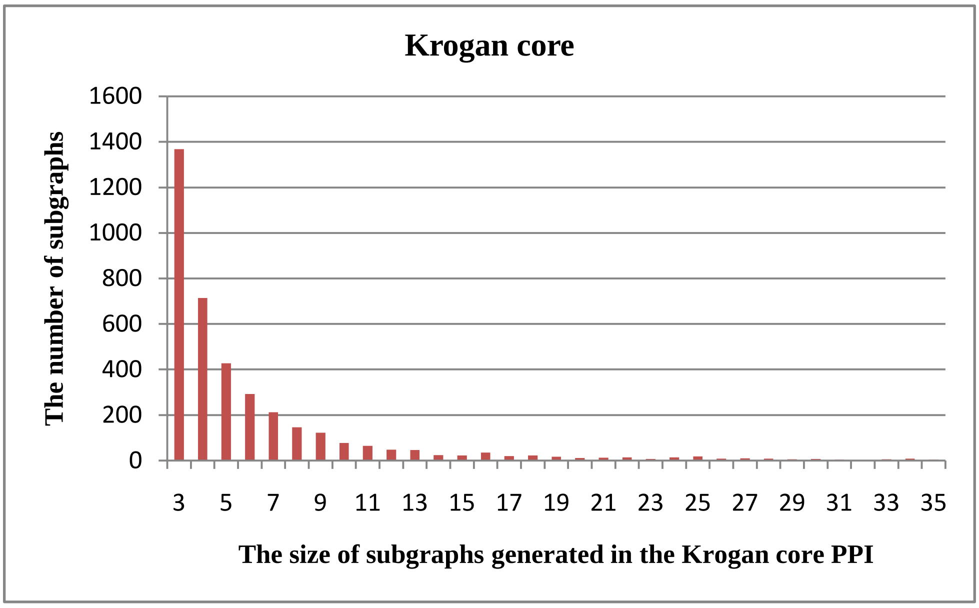

(e) Krogan core

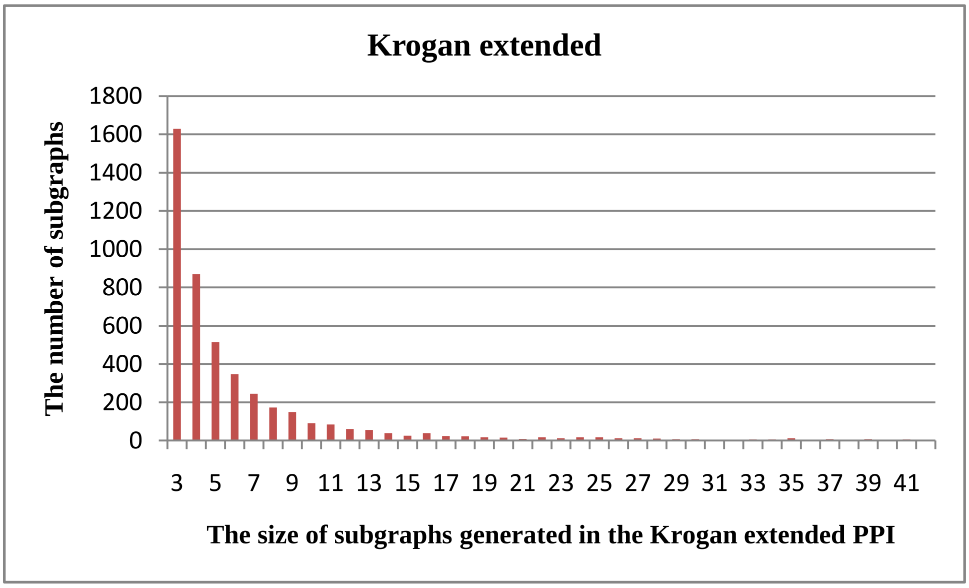

(f) Krogan extended

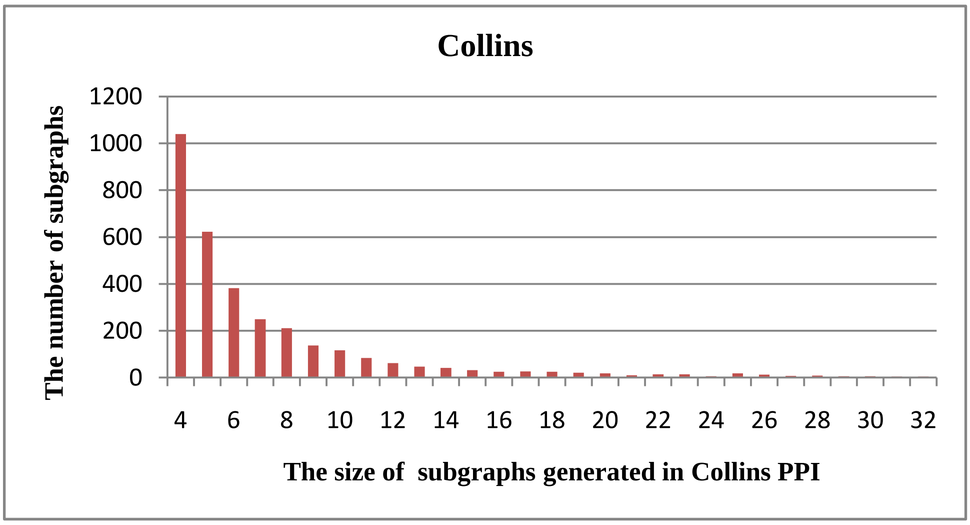

(g) Collins

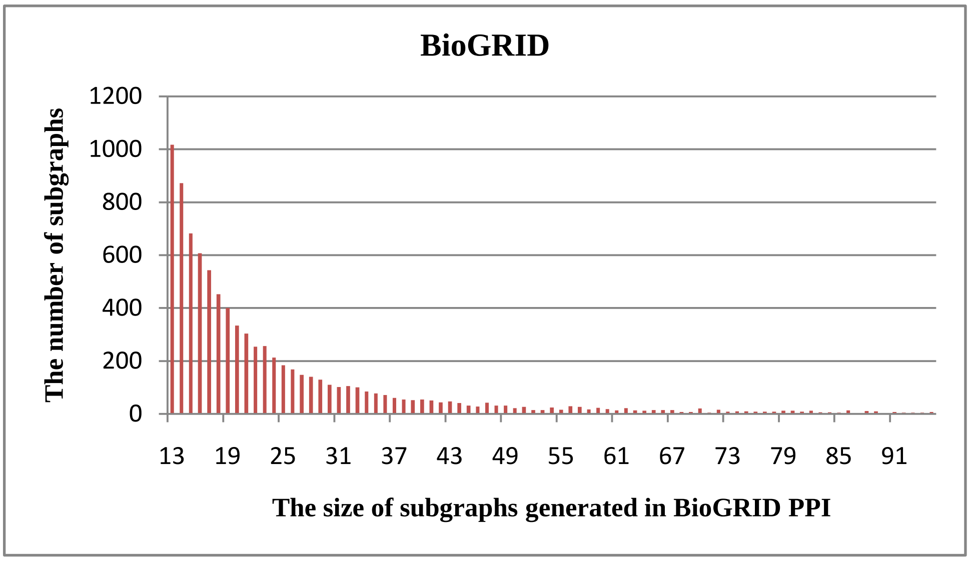

(h) BioGRID

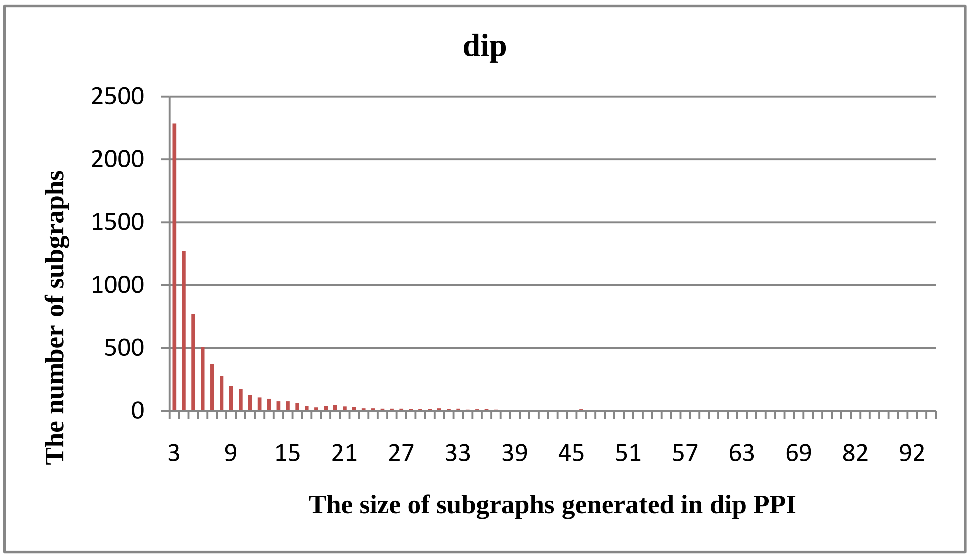

(i) dip

Supplementary Figure 2: The size distribution of true complexes and non-complex subgraphs

5 Parameter settings

5.1 Support threshold parameters setting for mining NEPs

Mining NEPs of complexes and non-complexes requires two support thresholds $\boldsymbol{\delta 1} > 0$ and $\boldsymbol{\delta 2} > 0$, respectively. Different thresholds $\boldsymbol{\delta 1}$ and $\boldsymbol{\delta 2}$ will generate different number of NEPs. NEPs generated for the given support thresholds should fulfill these properties such as discriminative power and simplicity[2-3].

Given a PPI network, the instances constructed by true complexes in the PPI network are regarded as the positive class, while the instances constructed by non-complexes (random subgraphs) in PPI are considered as the negative class. In our experiments, the minimum support threshold in the complexes and the maximum support threshold in the non-complexes for mining NEPs of complexes; the minimum

support threshold in the non-complexes and the maximum support threshold in the complexes for mining NEPs of non-complexes in different datasets are shown in **the Supplementary Tables** 5-10.

Supplementary Table 5：The support threshold for mining NEPs in the Gavin dataset

	Minimum support in the complexes	Maximum support in the non-complexes	Maximum support in the complexes	Minimum support in the non- complexes
NEPs of complexes	0.4	0.05		
NEPs of non-complexes			0.05	0.4

Supplementary Table 6：The support threshold for mining NEPs in the Krogan core dataset

	Minimum support in the complexes	Maximum support in the non-complexes	Maximum support in the complexes	Minimum support in the non- complexes
NEPs of complexes	0.4	0.05		
NEPs of non-complexes			0.05	0.4

Supplementary Table 7：The support threshold for mining NEPs in the Krogan extended dataset

	Minimum support in the complexes	Maximum support in the non-complexes	Maximum support In the complexes	Minimum support in the non- complexes
NEPs of complexes	0.4	0.05		
NEPs of non-complexes			0.05	0.4

Supplementary Table 8：The support threshold for mining NEPs in the BioGRID dataset

	Minimum support in the complexes	Maximum support in the non-complexes	Maximum support in the complexes	Minimum support in the non- complexes
NEPs of complexes	0.4	0.05		
NEPs of non-complexes			0.05	0.4

Supplementary Table 9：The support threshold for mining NEPs in the Collins dataset

	Minimum support in the complexes	Maximum support in the non-complexes	Maximum support in the complexes	Minimum support in the non- complexes
NEPs of complexes	0.6	0.04		
NEPs of non-complexes			0.03	0.3

Supplementary Table 10: The support threshold for mining NEPs in the DIP dataset

	Minimum support in the complexes	Maximum support in the non-complexes	Maximum support in the complexes	Minimum support in the non- complexes
NEPs of complexes	0.4	0.05		
NEPs of non-complexes			0.05	0.3

5.2 Parameter settings of ClusterEPs and other tested algorithms

During the process of searching complexes by ClusterEPs, the merging threshold was set as 0.9. The size threshold of the cluster was set as 100 when searching complexes. We did not tune these parameters to a particular dataset in our experiments.

Except ClusterONE, parameters in other six tested algorithms have been preliminarily optimized to a specific dataset in order to obtain the best possible results. These optimized parameters were obtained by trying all possible combinations in [4]. In our experiments, we used the optimized parameter values which were tested for each algorithm on each dataset in [4].

6 Mined NEPs in each dataset

NEPs mined in each PPI network are listed in the Supplementary Tables 11-16.

Supplementary Table 11: NEPs in the Gavin PPI network

NEPs	Support (%) in complexes	Support(%) in non-complexes
{*graphDensity* > 0.9}	56.7	0
{*meanClusteringCoeff* > 0.9}	57.5	0
{*meanTopologicCoeff* > 0.9}	59.7	0
{*varDegreeCorrelation* <= 0.42}	64.2	0
{*maxTopologicCoeff* > 0.9}	77.6	0.7
{*maxClusteringCoeff* > 0.9}	88.8	0.7
{1.7 < *maxDegree* <= 3.4}	45.5	1.4
{1.6<*maxDegreeCorrelation* <= 3.27}	44.5	1.4
{*eigenValue_2* <= 0.22}	1.5	80.4
{*graphDensity* <= 0.1}	1.5	99.7

Supplementary Table 12：NEPs in the Krogan core PPI network

NEPs	Support (%) in complexes	Support(%) in non-complexes
{*maxTopologicCoeff* > 0.9}	46.8	0.3
{*maxClusteringCoeff* > 0.9}	68.4	0.3
{0.8< *eigenValue*_2 <= 1.6, *nodeSize* <=6.2}	59.1	1.5
{1.55 < *meanDegreeCorrelation* <= 3.1}	48.0	1.1
{1.77 < *maxDegreeCorrelation* <= 3.5}	47.4	1.3
{*graphDensity* <= 0.1 }	1.8	98.4
{ *eigenValue*_2 <=0.81, 324.4 < *aveLength* <= 532.7}	2.3	40.5
{ *eigenValue*_2 <=0.81, 364.9< *aveWeight* <= 604.1}	2.9	40.8
{*maxDegreeCorrelation* <= 1.77, 324.4 < *aveLength* <= 532.7}	2.9	43.1
{ *maxDegreeCorrelation* <= 1.77, 364.9< *aveWeight* <= 604.1}	3.5	43.5
{*meanDegreeCorrelation*<=1.55, 324.4 < *aveLength* <= 532.7}	2.9	43.3
{ *meanDegreeCorrelation*<=1.55, 364.9< *aveWeight* <= 604.1}	3.5	43.6

Supplementary Table 13：NEPs in the Krogan extended PPI network

NEPs	Support (%) in complexes	Support(%) in non-complexes
{*varDegreeCorrelation* <= 0.53}	50.2	0
{*maxTopologicCoeff* > 0.9}	52.7	0.1
{*maxClusteringCoeff* > 0.9}	67.3	0.2
{0.81 <*eigenValue*_2 <= 1.6}	60.5	5.0
{1.6 <*meanDegreeCorrelation* <=3.2}	46.8	1.0
{1.85<*maxDegreeCorrelation*<=3.7}	44.4	1.6
{ *graphDensity* <=0.1, *eigenValue*_2<= 0.8}	3.9	94.2
{ *graphDensity* <=0.1, 0.53<*varDegreeCorrelation*<= 1.1}	3.4	89.9
{ *graphDensity* <=0.1, *maxDegreeCorrelation*<= 1.85}	4.4	97.1
{ *graphDensity* <=0.1, *maxTopologicCoeff*<= 0.1}	4.9	98.2
{ *graphDensity* <=0.1, *meanTopologicCoeff*<= 0.1}	4.9	98.5
{ *graphDensity* <=0.1, *maxClusteringCoeff*<= 0.1}	4.9	98.4
{ *graphDensity* <=0.1, *meanClusteringCoeff*<= 0.1}	4.9	98.5
{ *graphDensity* <=0.1, eigenValue_1<= 1.46}	4.9	98.5
{ *graphDensity* <=0.1, eigenValue_3<= 0.31}	3.9	97.1
{ *graphDensity* <=0.1, *varClusteringCoeff*<= 0.05}	4.9	98.4
{*graphDensity* <=0.1, *nodeSize*<= 6.2 }	3.9	73.0
{ *eigenValue*_2<= 0.8, 0.53<*varDegreeCorrelation*<= 1.1}	3.4	89.9
{0.53<*varDegreeCorrelation*<=1.1,*maxDegreeCorrelation*<= 1.85 }	3.9	89.9
{0.53<*varDegreeCorrelation*<=1.1,*meanDegreeCorrelation*<= 1.61}	4.4	89.9
{ 0.53<*varDegreeCorrelation*<= 1.1, *eigenValue*_1<= 1.46}	4.4	89.9
{0.53<*varDegreeCorrelation*<= 1.1, *maxDegree*<= 2.1 }	3.9	89.9

Supplementary Table 14：NEPs in the Collins PPI network

NEPs	Support (%) in complexes	Support(%) in non-complexes
{*meanClusteringCoeff* >0.9}	60.8	0
{*meanTopologicCoeff* > 0.9}	60.8	0
{*varDegreeCorrelation* < = 0.55}	79.1	0
{*maxTopologicCoeff* > 0.9}	78.5	1.9
{*maxClusteringCoeff* > 0.9}	89.2	2.4
{*graphDensity* < = 0.1}	0.6	97.5
{0.55 < *varDegreeCorrelation*<=1.1, *eigenValue_*3<=0.31}	2.5	78.0
{0.55<*varDegreeCorrelation*<=1.1, *maxTopologicCoeff* <= 0.1}	2.5	78.0
{0.55<*varDegreeCorrelation*<=1.1, *maxClusteringCoeff* <= 0.1}	2.5	78.0
{0.55<*varDegreeCorrelation*<=1.1, *meanTopologicCoeff* <= 0.1}	2.5	78.0
{0.55<*varDegreeCorrelation*<=1.1, *meanClusteringCoeff* <= 0.1}	2.5	78.0
{0.55<*varDegreeCorrelation*<=1.1, *maxDegree* <= 2.4}	0.6	78.0
{0.55<*varDegreeCorrelation*<=1.1, *maxDegreeCorrelation* < =2.2}	0.6	78.0
{0.55<*varDegreeCorrelation*<=1.1, *meanDegreeCorrelation* <= 2.0 }	1.3	78.0
{0.55<*varDegreeCorrelation*<=1.1, *nodeSize*<=5.9}	2.5	47.2
{0.55<*varDegreeCorrelation*<=1.1, *varClusteringCoeff*<=0.05}	2.5	78.0
{0.55<*varDegreeCorrelation*<=1.1, *eigenValue_*2 <= 1.26}	2.5	78.0
{0.55<*varDegreeCorrelation*<=1.1, *meanDegree* <= 1.9, *varTopologicCoeff*<=0.05}	2.5	78.0
{0.55<*varDegreeCorrelation*<=1.1, *eigenValue_*1 <= 2.1, *varTopologicCoeff*<=0.05}	2.5	78.0
{0.55<*varDegreeCorrelation*<=1.1, *medianDegree* <= 2.2, *varTopologicCoeff*<=0.05}	2.5	78.0
{0.55<*varDegreeCorrelation*<=1.1, *varTopologicCoeff* <=0.05, *varDegree*<= 4.15 }	2.5	78.0

Supplementary Table 15：NEPs in the BioGRID PPI network

NEPs	Support (%) in complexes	Support(%) in non-complexes
{*graphDensity* > 0.9}	50.3	0
{*meanTopologicCoeff* > 0.9}	53.8	0
{*meanClusteringCoeff* > 0.9}	54.0	0
{*varDegreeCorrelation*<=0.67 }	71.3	0
{*maxTopologicCoeff* > 0.9}	69.8	2.0
{*maxClusteringCoeff* > 0.9}	87.8	3.8
{0.83<*eigenValue*_3<=1.66, *nodeSize*<= 12.2}	69.8	1.5
{0.83<*eigenValue*_3<=1.66, *eigenValue*_2<=1.07 }	48.3	4.8
{*graphDensity*<= 0.1}	4.3	97.7
{*eigenValue*_3<= 0.83, *eigenValue*_2<=1.1}	4.5	86.0
{ *eigenValue*_3<= 0.83, 0.672<*varDegreeCorrelation*<=1.34, *meanDegreeCorrelation*<=2.383}	4.3	71.5
{*maxTopologicCoeff*<= 0.1, 433.3<*aveLength*<= 603.4}	1.3	40.3
{*maxTopologicCoeff*<= 0.1, 494.6 <*aveWeight*<= 690.2}	1.3	40.1
{*maxTopologicCoeff*<= 0.1, *eigenValue*_2<= 1.1}	5.0	90.0
{*maxTopologicCoeff*<= 0.1, 0.672<*varDegreeCorrelation*<=1.34, *meanDegreeCorrelation*<=2.383}	4.3	71.8
{*meanTopologicCoeff* <= 0.1, 433.3<*aveLength*<= 603.4}	1.3	43.2
{*meanTopologicCoeff* <= 0.1, 494.6 <*aveWeight*<= 690.2}	1.3	43.0
{*meanTopologicCoeff* <= 0.1, *eigenValue*_2<=1.1}	5.0	90.2
{*meanTopologicCoeff* <= 0.1, 0.672<*varDegreeCorrelation*<=1.34, *meanDegreeCorrelation*<=2.383}	4.3	71.8
{0.672<*varDegreeCorrelation*<=1.34, *eigenValue*_2<=1.1}	4.3	71.8
{0.672<*varDegreeCorrelation*<=1.34,*maxClusteringCoeff*<= 0.1, *meanDegreeCorrelation*<=2.383}	4.5	71.8
{0.672<*varDegreeCorrelation*<=1.34,*meanClusteringCoeff*<=0.1 *meanDegreeCorrelation*<=2.4}	4.5	71.8
{0.672<*varDegreeCorrelation*<=1.34,*meanDegreeCorrelation*<=2.4 ,*eigenValue*_1<=2.4}	5.0	71.8
{0.672<*varDegreeCorrelation*<=1.34,*meanDegreeCorrelation*<=2.4 ,*varClusteringCoeff*<= 0.05}	4.5	71.8
{0.672<*varDegreeCorrelation*<=1.34,*meanDegreeCorrelation*<=2.4 ,*varTopologicCoeff*<=0.05}	4.3	71.8
{*maxClusteringCoeff*<= 0.1, 433.3<*aveLength*<= 603.4}	1.3	41.6
{*maxClusteringCoeff*<= 0.1, 494.6 <*aveWeight*<= 690.2}	1.3	41.5
{*maxClusteringCoeff*<= 0.1, *eigenValue*_2<=1.1}	5.0	90.0
{ *meanClusteringCoeff*<=0.1,433.3<*aveLength*<= 603.4}	1.3	43.7
{ *meanClusteringCoeff*<=0.1, 494.6 <*aveWeight*<= 690.2}	1.3	43.5
{ *meanClusteringCoeff*<=0.1, *eigenValue*_2<=1.1}	5.0	90.2

Supplementary Table 16：NEPs in the CombinedYeast PPI network

NEPs	Support (%) in complexes	Support(%) in non-complexes
{0.93<*meanTopologicCoeff*><=1.06}	42.2	0
{0.9<*graphDensity*<=1.05}	43.1	0
{0.94<*meanClusteringCoeff*<=1.092}	44.7	0
{*nodeSize*<=12.2}	81.5	0
{0.8<*maxClusteringCoeff* <=1}	55.0	2.7
{0.9<*maxTopologicCoeff*<= 1.05, *varTopologicCoeff*<= 0.054 }	41.2	0
{0.9<*maxTopologicCoeff*<= 1.05, *varClusteringCoeff*<= 0.09}	40.0	0
{*graphDensity*<= 0.15, *maxTopologicCoeff*<= 0.15}	3.5	79.7
{*graphDensity*<= 0.15, *maxClusteringCoeff*<= 0.2}	3.7	81.4
{*graphDensity*<= 0.15, *meanClusteringCoeff*<= 0.156 }	3.7	99.2
{*graphDensity*<= 0.15, *meanTopologicCoeff*<= 0.133}	3.7	99.5
{*graphDensity*<= 0.15, 12.2 <nodeSize<= 21.4}	1.4	61.0
{*graphDensity*<= 0.15, *eigenValue*_3<= 0.866}	2.6	57.7
{*graphDensity*<= 0.15, 433.27 <*aveLength*<= 603.4 }	3.0	64.4
{*graphDensity*<= 0.15, 494.6 <*aveWeight*<= 690.2 }	3.0	63.2
{*graphDensity*<= 0.15, *meanDegreeCorrelation*<=3.762 }	4.7	98.9
{*graphDensity*<= 0.15, *varClusteringCoeff*<= 0.09}	3.7	81.7
{*graphDensity*<= 0.15, *varTopologicCoeff*<=0.054}	3.5	79.8
{*graphDensity*<= 0.15, *maxDegreeCorrelation*<= 4.9}	4.7	97.7
{*graphDensity*<= 0.15, 0.866<*eigenValue*_3<=1.732 }	2.6	40.4
{*graphDensity*<= 0.15, varDegreeCorrelation<= 2.346}	4.2	88.4
{*maxTopologicCoeff*<= 0.15, 12.2 <*nodeSize*<= 21.4}	0.5	55.6
{*maxTopologicCoeff*<= 0.15, 433.27 <*aveLength*<= 603.4}	2.6	49.2
{*maxTopologicCoeff*<= 0.15, 494.6 <*aveWeight*<= 690.2 }	2.6	48.5
{*maxClusteringCoeff*<= 0.2, 12.2 <*nodeSize*<= 21.4}	0.5	55.8
{ *maxClusteringCoeff*<= 0.2, 433.27 <*aveLength*<= 603.4}	2.6	50.6
{ *maxClusteringCoeff*<= 0.2, 494.6 <*aveWeight*<= 690.2}	2.6	49.7
{ *meanClusteringCoeff*<= 0.156, 12.2 <*nodeSize*<= 21.4}	0.5	60.5
{ *meanClusteringCoeff*<= 0.156, 433.27 <*aveLength*<= 603.4}	2.6	63.9
{ *meanClusteringCoeff*<= 0.156, 494.6 <*aveWeight*<= 690.2}	2.6	62.7
{ *meanTopologicCoeff*<= 0.133, 12.2 <*nodeSize*<= 21.4}	0.5	60.6
{ *meanTopologicCoeff*<= 0.133, 433.27 <*aveLength*<= 603.4}	2.6	64.1
{ *meanTopologicCoeff*<= 0.133, 494.6 <*aveWeight*<= 690.2}	2.6	62.9
{ *meanTopologicCoeff*<= 0.133, 0.866<*eigenValue*_3<=1.732}	1.9	40.1
{ 12.2 <*nodeSize*<= 21.4, *eigenValue*_3<= 0.866}	0.2	47.1
{ 12.2 <*nodeSize*<= 21.4, *meanDegree*<= 2.4 }	1.9	61.0
{ 12.2 <*nodeSize*<= 21.4, *meanDegreeCorrelation*<= 3.762 }	1.6	60.8
{ 12.2 <*nodeSize*<= 21.4, *medianDegree*<= 3 }	1.9	61.0
{ 12.2 <*nodeSize*<= 21.4, *eigenValue*_1<= 3.896 }	1.6	61.0
{ 12.2 <*nodeSize*<= 21.4, *varClusteringCoeff*<= 0.09 }	4.7	55.8
{ 12.2 <*nodeSize*<= 21.4, *maxDegreeCorrelation*<= 4.9 }	1.4	60.8
{ 12.2 <nodeSize<= 21.4, *maxDegree*<= 5.1 }	1.4	60.8

7 Quality scores of the predicted complexes

7.1 Results on five yeast PPI Datasets

Our experiments were conducted on two different personal computers. The specifications of these two personal computers (PC) are provided as follows:

(1) PC1: 4 Intel(R) Core(TM) i5-3230 CPU, 2.6GHz each and 8GB of RAM. The Operation System : window 8.

(2) PC2: 8 Intel(R) Core(TM) i7-3770 CPU, 3.4GHz each and 4GB of RAM. The Operation System is window 7.

We ran ClusterEPs 10 times in each personal computer for each dataset. The results obtained using MIPS and SGD on five datasets are shown in the **Supplementary Tables** 17-26. The first column of each table denotes the personal computers used in experiments. The second column of each table denotes the experiment ID in each personal computer. Columns 4-6 respectively denotes three quality scores. The rightmost column is the sum of three quality scores.

In the **Supplementary Tables** 17-26, the second row starting from the bottommost of each table denotes the average value of each quality score. The last row of each table denotes the Standard variance of 20 values for each quality score.

Supplementary Table 17: Results obtained using MIPS as the test set on the Gavin dataset

PC	ID	#cluster	Fra	Acc	MMR	Composite score
PC1	1	241	0.7652	0.4752	0.426	1.6664
	2	241	0.7652	0.4752	0.426	1.6664
	3	241	0.7652	0.4752	0.426	1.6664
	4	240	0.7739	0.4765	0.4288	1.6792
	5	240	0.7739	0.4765	0.4288	1.6792
	6	241	0.7652	0.4752	0.426	1.6664
	7	241	0.7652	0.4752	0.426	1.6664
	8	240	0.7739	0.4765	0.4288	1.6792
	9	237	0.7652	0.4754	0.4266	1.6672
	10	241	0.7652	0.4752	0.426	1.6664
PC2	1	237	0.7739	0.4824	0.4285	1.6848
	2	241	0.7826	0.4817	0.435	1.6993
	3	240	0.7826	0.4817	0.435	1.6993
	4	236	0.7739	0.482	0.4314	1.6873
	5	241	0.7826	0.4817	0.435	1.6993
	6	241	0.7826	0.4817	0.435	1.6993
	7	239	0.7826	0.4821	0.4379	1.7026
	8	240	0.7826	0.4817	0.435	1.6993
	9	240	0.7826	0.4817	0.435	1.6993
	10	240	0.7826	0.4817	0.435	1.6993
	Average	240	0.774	0.479	0.431	1.684
	Standard variance	1.518	0.008	0.003	0.004	0.015

Supplementary Table 18： Results obtained using SGD as the test set on the Gavin dataset

PC	ID	#cluster	Fra	Acc	MMR	Composite score
PC1	1	245	0.843	0.657	0.5325	2.0325
	2	245	0.843	0.657	0.5325	2.0325
	3	247	0.843	0.6558	0.532	2.0308
	4	245	0.843	0.657	0.5325	2.0325
	5	245	0.8512	0.656	0.5306	2.0378
	6	245	0.843	0.657	0.5325	2.0325
	7	249	0.843	0.656	0.532	2.031
	8	249	0.843	0.656	0.532	2.031
	9	242	0.8512	0.6569	0.5312	2.0393
	10	249	0.843	0.656	0.532	2.031
PC2	1	241	0.8017	0.6575	0.5135	1.9727
	2	241	0.8017	0.6575	0.5135	1.9727
	3	241	0.8017	0.6575	0.5135	1.9727
	4	244	0.8099	0.6592	0.5247	1.9938
	5	244	0.8099	0.6592	0.5247	1.9938
	6	244	0.8099	0.6592	0.5247	1.9938
	7	244	0.8099	0.6592	0.5247	1.9938
	8	241	0.8017	0.6575	0.5135	1.9727
	9	244	0.8099	0.6592	0.5247	1.9938
	10	241	0.8017	0.6575	0.5135	1.9727
Average		244	0.825	0.657	0.526	2.008
Standard variance		2.677	0.020	0.001	0.0078	0.027

Supplementary Table 19： Results obtained using MIPS as the test set on the Krogan core dataset

PC	ID	#cluster	Fra	Acc	MMR	Composite score
PC1	1	385	0.694	0.4175	0.3704	1.4819
	2	373	0.6716	0.4196	0.3601	1.4513
	3	372	0.6567	0.4172	0.3525	1.4264
	4	372	0.6567	0.4172	0.3525	1.4264
	5	375	0.6567	0.4164	0.3525	1.4256
	6	375	0.6567	0.4164	0.3525	1.4256
	7	372	0.6567	0.4172	0.3525	1.4264
	8	372	0.6567	0.4172	0.3525	1.4264
	9	372	0.6567	0.4172	0.3525	1.4264
	10	372	0.6567	0.4172	0.3525	1.4264
PC2	1	354	0.7239	0.4235	0.3585	1.5059
	2	355	0.7164	0.4236	0.3583	1.4983
	3	354	0.7239	0.4235	0.3585	1.5059
	4	353	0.7164	0.4224	0.3557	1.4945
	5	362	0.7015	0.4225	0.3598	1.4838
	6	354	0.7239	0.4233	0.3585	1.5057
	7	354	0.7239	0.4233	0.3585	1.5057
	8	354	0.7239	0.4233	0.3585	1.5057
	9	354	0.7239	0.4233	0.3585	1.5057
	10	354	0.7239	0.4233	0.3585	1.5057
Average		364	0.691	0.420	0.357	1.468
Standard variance		10.389	0.031	0.003	0.004	0.037

Supplementary Table 20： Results obtained using SGD as the test set on the Krogan core dataset

PC	ID	#cluster	Fra	Acc	MMR	Composite score
PC1	1	278	0.6478	0.5858	0.4386	1.6722
	2	304	0.6478	0.5821	0.4282	1.6581
	3	280	0.6541	0.5848	0.4419	1.6808
	4	322	0.6667	0.5831	0.4502	1.7
	5	322	0.6667	0.5877	0.4424	1.6968
	6	322	0.6667	0.5845	0.4502	1.7014
	7	285	0.6352	0.5863	0.4328	1.6543
	8	290	0.6289	0.5818	0.4293	1.64
	9	322	0.6604	0.5879	0.4422	1.6905
	10	326	0.6855	0.5872	0.4581	1.7308
PC2	1	256	0.6164	0.58	0.4129	1.6093
	2	281	0.6352	0.5858	0.4223	1.6433
	3	277	0.6226	0.5828	0.4163	1.6217
	4	279	0.6226	0.5841	0.4194	1.6261
	5	281	0.6352	0.5858	0.4223	1.6433
	6	257	0.6164	0.58	0.4134	1.6098
	7	284	0.6415	0.5811	0.4242	1.6468
	8	273	0.6101	0.5781	0.411	1.5992
	9	325	0.6541	0.5796	0.4401	1.6738
	10	280	0.6226	0.5841	0.4189	1.6256
Average		292.2	0.642	0.584	0.431	1.656
Standard variance		23.064	0.021	0.003	0.014	0.036

Supplementary Table 21： Results obtained using MIPS as the test set on the Krogan extended dataset

PC	ID	#cluster	Fra	Acc	MMR	Composite score
PC1	1	549	0.6	0.3873	0.3123	1.2996
	2	549	0.6	0.3875	0.3123	1.2998
	3	549	0.6	0.3873	0.3123	1.2996
	4	567	0.6	0.3847	0.3179	1.3026
	5	640	0.5806	0.3835	0.3082	1.2723
	6	549	0.6	0.3873	0.3123	1.2996
	7	627	0.5677	0.3837	0.3037	1.2551
	8	549	0.6	0.3873	0.3123	1.2996
	9	549	0.6	0.3873	0.3123	1.2996
	10	642	0.5806	0.3841	0.3086	1.2733
PC2	1	543	0.6065	0.3864	0.33	1.3229
	2	543	0.6065	0.3864	0.33	1.3229
	3	537	0.6	0.3871	0.3207	1.3078
	4	543	0.6065	0.3864	0.33	1.3229
	5	543	0.6065	0.3864	0.33	1.3229
	6	533	0.5935	0.3849	0.3239	1.3023
	7	529	0.6	0.3858	0.3224	1.3082
	8	528	0.6	0.3863	0.3224	1.3087
	9	543	0.6065	0.3864	0.33	1.3229
	10	617	0.6	0.3852	0.3215	1.3067
	Average	561	0.598	0.386	0.319	1.302
	Standard variance	37.193	0.010	0.0013	0.009	0.0181

Supplementary Table 22: Results obtained using SGD as the test set on the Krogan extended dataset

PC	ID	#cluster	Fra	Acc	MMR	Composite score
PC1	1	568	0.7314	0.5539	0.4374	1.7227
	2	558	0.7143	0.5555	0.4406	1.7104
	3	510	0.6571	0.5519	0.4202	1.6292
	4	522	0.6914	0.5602	0.4298	1.6814
	5	517	0.6686	0.5586	0.4262	1.6534
	6	501	0.6571	0.5517	0.4224	1.6312
	7	558	0.7143	0.5553	0.4406	1.7102
	8	517	0.6857	0.5591	0.429	1.6738
	9	551	0.7143	0.5521	0.4385	1.7049
	10	552	0.7086	0.5529	0.4396	1.7011
PC2	1	505	0.6343	0.5434	0.3929	1.5706
	2	501	0.64	0.5463	0.3941	1.5804
	3	486	0.64	0.5432	0.3969	1.5801
	4	506	0.6514	0.5474	0.3985	1.5973
	5	508	0.6514	0.5462	0.3982	1.5958
	6	493	0.6343	0.5401	0.3891	1.5635
	7	513	0.6457	0.5428	0.3947	1.5832
	8	552	0.6514	0.5501	0.4009	1.6024
	9	517	0.6457	0.5436	0.3945	1.5838
	10	506	0.64	0.5439	0.3953	1.5792
Average		522	0.669	0.550	0.414	1.633
Standard variance		24.778	0.032	0.006	0.020	0.056

Supplementary Table 23: Results obtained using MIPS as the test set on the Collins dataset

PC	ID	#cluster	Fra	Acc	MMR	Composite score
PC1	1	176	0.7043	0.5189	0.428	1.6512
	2	176	0.7043	0.5189	0.428	1.6512
	3	176	0.7043	0.5189	0.428	1.6512
	4	176	0.7043	0.5189	0.428	1.6512
	5	176	0.7043	0.5189	0.428	1.6512
	6	176	0.7043	0.5189	0.428	1.6512
	7	176	0.7043	0.5189	0.428	1.6512
	8	176	0.7043	0.5189	0.428	1.6512
	9	176	0.7043	0.5189	0.428	1.6512
	10	176	0.7043	0.5189	0.428	1.6512
PC2	1	167	0.7043	0.5381	0.4086	1.651
	2	166	0.713	0.5376	0.4161	1.6667
	3	167	0.7043	0.5381	0.4086	1.651
	4	166	0.713	0.5376	0.4161	1.6667
	5	166	0.6957	0.5382	0.4116	1.6455
	6	166	0.713	0.5377	0.4185	1.6692
	7	166	0.7043	0.5385	0.4168	1.6596
	8	166	0.6957	0.538	0.4091	1.6428
	9	167	0.7043	0.5381	0.4086	1.651
	10	166	0.713	0.5376	0.4161	1.6667
	Average	171	0.705	0.528	0.421	1.654
	Standard variance	4.987	0.005	0.009	0.008	0.007

Supplementary Table 24：Results obtained using SGD as the test set on the Collins datasets

PC	ID	#cluster	Fra	Acc	MMR	Composite score
PC1	1	382	0.7786	0.646	0.4981	1.9227
	2	502	0.7863	0.6188	0.5066	1.9117
	3	423	0.7863	0.6382	0.5213	1.9458
	4	427	0.7939	0.6393	0.5222	1.9554
	5	514	0.8092	0.6206	0.5121	1.9419
	6	326	0.8168	0.6306	0.5472	1.9946
	7	422	0.8168	0.6515	0.5269	1.9952
	8	417	0.8168	0.6498	0.5208	1.9874
	9	242	0.771	0.6437	0.5133	1.928
	10	346	0.7939	0.658	0.5123	1.9642
PC2	1	341	0.8092	0.6615	0.5531	2.0238
	2	422	0.7863	0.633	0.5302	1.9495
	3	259	0.8015	0.6521	0.562	2.0156
	4	408	0.8321	0.6529	0.5642	2.0492
	5	363	0.8168	0.6429	0.5565	2.0162
	6	422	0.8244	0.6506	0.5474	2.0224
	7	414	0.8321	0.652	0.5531	2.0372
	8	415	0.8168	0.653	0.546	2.0158
	9	365	0.8168	0.6603	0.5613	2.0384
	10	290	0.8015	0.6599	0.5589	2.0203
Average		385	0.805	0.646	0.536	1.987
Standard variance		70.382	0.0176	0.012	0.021	0.043

Supplementary Table 25: Results obtained using MIPS as the test set on the BioGRID dataset

PC	ID	#cluster	Fra	Acc	MMR	Composite score
PC1	1	939	0.6082	0.3487	0.2857	1.2426
	2	939	0.6082	0.3487	0.2857	1.2426
	3	939	0.6082	0.3486	0.2857	1.2425
	4	934	0.6082	0.35	0.2869	1.2451
	5	934	0.6082	0.35	0.2869	1.2451
	6	934	0.6082	0.3501	0.2869	1.2452
	7	939	0.6082	0.3487	0.2857	1.2426
	8	934	0.6082	0.35	0.2869	1.2451
	9	938	0.6082	0.3487	0.2857	1.2426
	10	698	0.6289	0.4129	0.3075	1.3493
PC2	1	876	0.6546	0.3578	0.312	1.3244
	2	660	0.6701	0.4157	0.3335	1.4193
	3	876	0.6546	0.3578	0.312	1.3244
	4	871	0.6546	0.3585	0.3136	1.3267
	5	655	0.6649	0.4185	0.3349	1.4183
	6	871	0.6546	0.3585	0.3136	1.3267
	7	888	0.6598	0.3571	0.3167	1.3336
	8	876	0.6546	0.3576	0.312	1.3242
	9	654	0.6649	0.4185	0.3351	1.4185
	10	876	0.6546	0.3578	0.312	1.3244
	Average	862	0.635	0.366	0.304	1.304
	Standard variance	104.039	0.026	0.026	0.018	0.064

Supplementary Table 26: Results obtained using SGD as the test set on the BioGRID dataset

PC	ID	#cluster	Fra	Acc	MMR	Composite score
PC1	1	831	0.6609	0.5192	0.3885	1.5686
	2	831	0.6609	0.5193	0.3885	1.5687
	3	821	0.6695	0.5212	0.3948	1.5855
	4	793	0.6781	0.5159	0.3901	1.5841
	5	790	0.6524	0.5417	0.3888	1.5829
	6	781	0.6609	0.543	0.3958	1.5997
	7	780	0.6524	0.5417	0.383	1.5771
	8	831	0.6609	0.5192	0.3885	1.5686
	9	653	0.6223	0.5119	0.3645	1.4987
	10	831	0.6609	0.5192	0.3885	1.5686
PC2	1	781	0.6824	0.5481	0.4126	1.6431
	2	783	0.6867	0.5493	0.4087	1.6447
	3	780	0.6867	0.5497	0.4147	1.6511
	4	782	0.6953	0.5553	0.3965	1.6471
	5	759	0.6738	0.5373	0.3948	1.6059
	6	741	0.6652	0.5659	0.4032	1.6343
	7	735	0.6609	0.5662	0.4077	1.6348
	8	737	0.6567	0.5643	0.4053	1.6263
	9	783	0.6867	0.5493	0.4087	1.6447
	10	777	0.6867	0.5492	0.4149	1.6508
	Average	780	0.668	0.539	0.397	1.604
	Standard variance	42.520	0.017	0.018	0.013	0.041

7.2 Discussion of the effect of random noise on quality scores

Supplementary Figure 3 shows an illustration of the relation among random subgraphs, unknown complexes and known complexes for a PPI network. In **Supplementary Figure 3**, let $\mathfrak{R}$ be the set of random subgraphs in a PPI network, and $\boldsymbol{\mathcal{K}}$ denote the set of known complexes which have been confirmed by the research community for a PPI network. Suppose $\boldsymbol{T}$ denote the set of true complexes contained in a PPI network. Obviously, $\boldsymbol{\mathcal{K}} \subseteq \boldsymbol{T}$ and $\boldsymbol{T} \subseteq \mathfrak{R}$. Let $\boldsymbol{\mathcal{U}} = \{\mathrm{u}|\mathrm{u} \in \boldsymbol{T}, \mathrm{u} \notin \boldsymbol{\mathcal{K}}\}$ and $\boldsymbol{\mathcal{N}} = \{\mathrm{n}|\mathrm{n} \in \mathfrak{R}, \mathrm{n} \notin \boldsymbol{\mathcal{K}}\}$, in other words, $\boldsymbol{\mathcal{U}}$ consists of true complexes which are unknown by biologists in the research community.

We use a supervised learning method to search complexes in ClusterEPs. In our method, the positive dataset was constructed by true complexes in $\boldsymbol{\mathcal{K}}$, and the negative dataset was constructed by randomly selecting non-complex subgraphs from $\boldsymbol{\mathcal{N}}$. Since $\boldsymbol{\mathcal{U}} \subseteq \boldsymbol{\mathcal{N}}$, a random subgraph would be likely selected from $\boldsymbol{\mathcal{U}}$. For a subgraph $\mathrm{s} \in \boldsymbol{\mathcal{U}}$, if s is selected to construct a negative instance, then we look on s as a non-complex, although s is a true complex. That is, s is regarded as the noise

(false negative). Although NEPs consider the potential noise, a large amount of noise will possibly affect the performance of ClusterEPs.

As more and more complexes are confirmed in the future, the number of complexes in $\mathcal{K}$ will become larger, the number of complexes in $\mathcal{U}$ will become less and less. This means the negative datasets would contain less and less noise, the performance of ClusterEPs would become better and better.

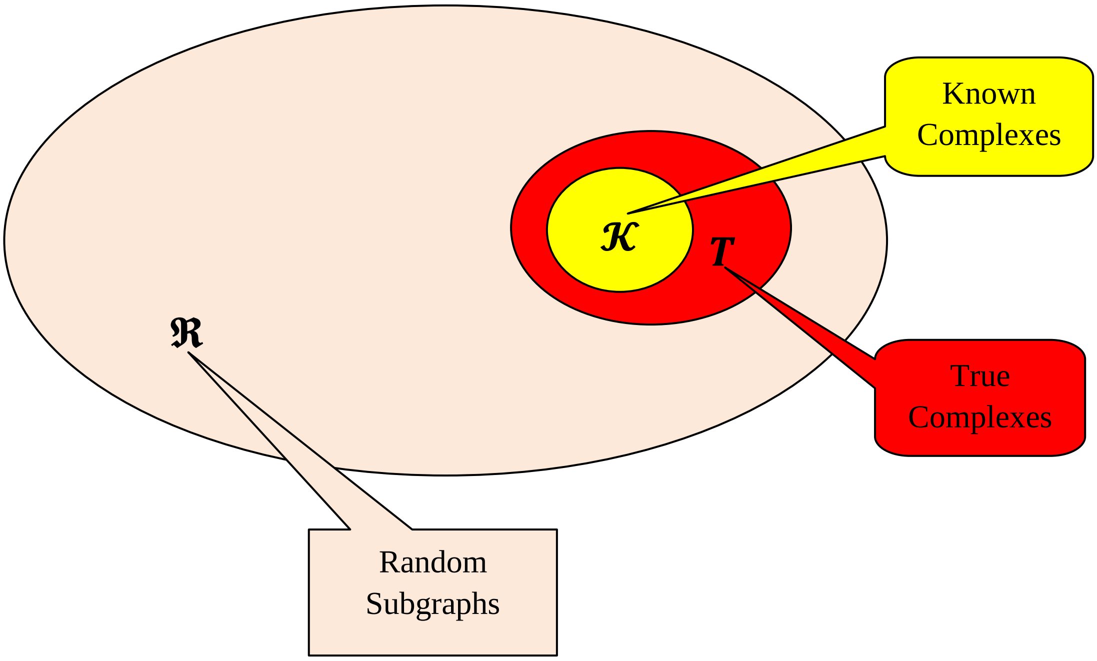

Supplementary Figure 3: An Illustration of the relation among random subgraphs, unknown complexes and known complexes for a PPI network.

8 Case studies

Supplementary Figure 4 presents the RNA polymerase I complex subgraph and its neighborhood subgraphs.

Supplementary Figure 5 presents the results of how the RNA polymerase I complex were identified by the eight algorithms. Only ClusterEPs was able to completely identify this complex with 100% precision. This subgraph is not a well-separated subgraph—it is connected to 109 external proteins outside the complex (see Supplementary Figure 5). This means that it has a higher boundary weight. Attributed to these facts, ClusterONE was not able to exactly recover this complex, but instead it added 10 nearby proteins almost to double the true size of the complex.

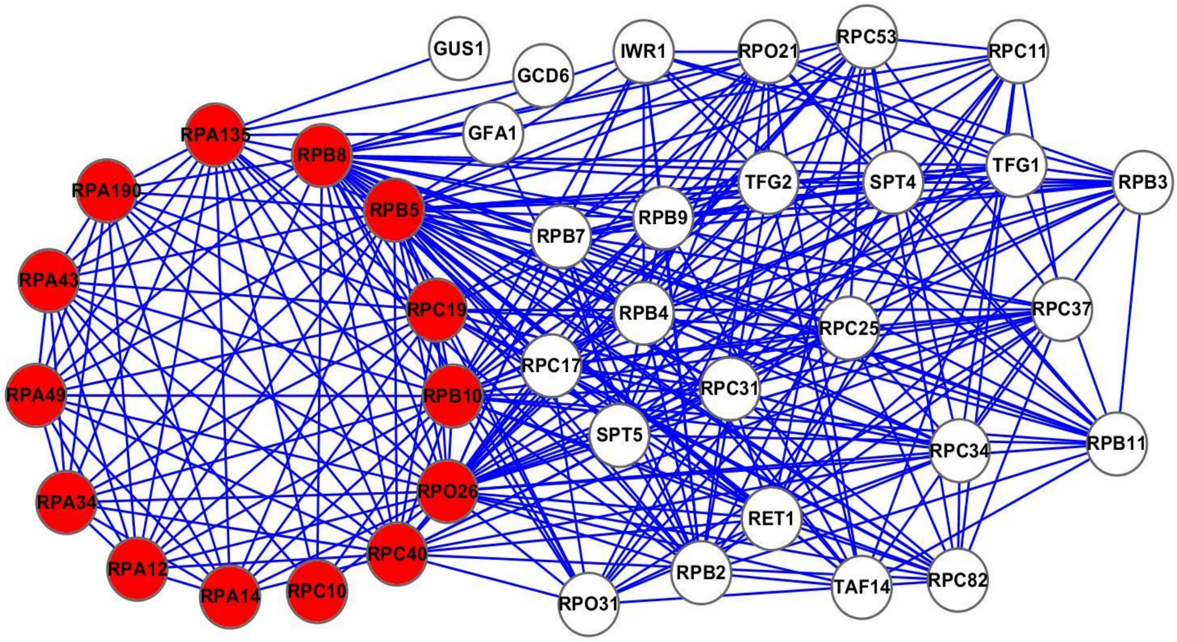

Supplementary Figure 4: The neighborhood subgraphs around the RNA polymerase I complex. Red nodes represent proteins that belong to the RNA polymerase I complex. White nodes represent neighbor proteins of the RNA polymerase I complex.

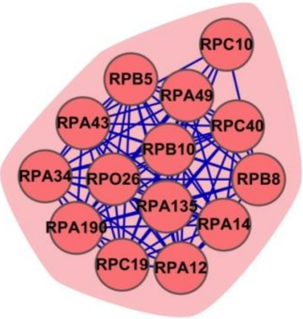

(a) ClusterEPs

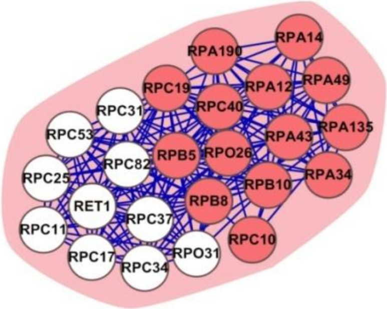

(b) ClusterONE

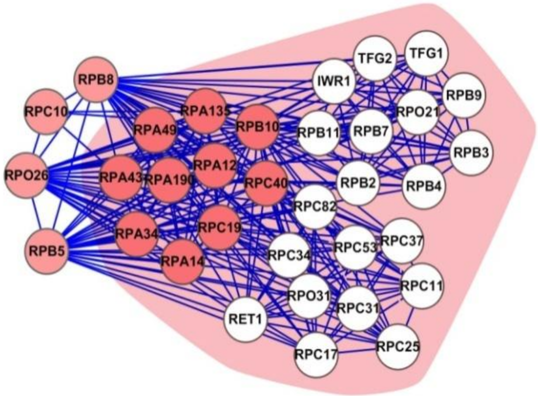

(c) MCODE

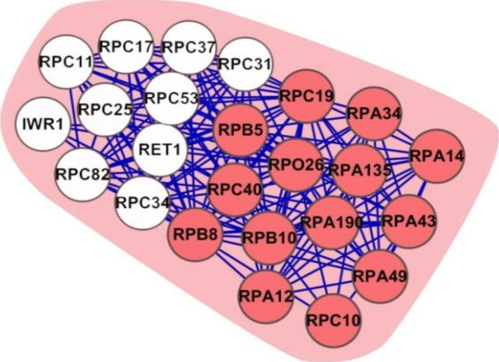

(d) MCL

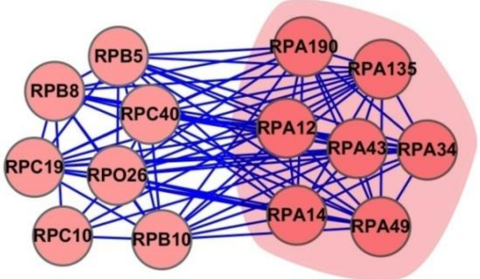

(e) RNSC

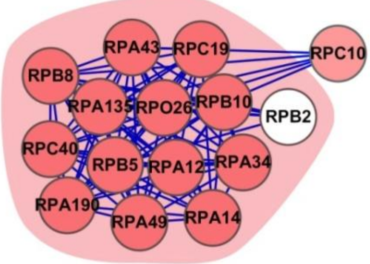

(f) CMC

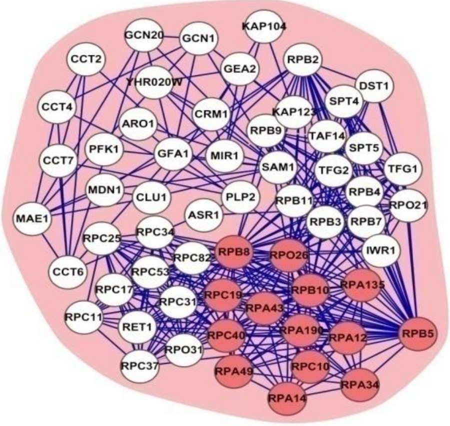

(g) CFinder

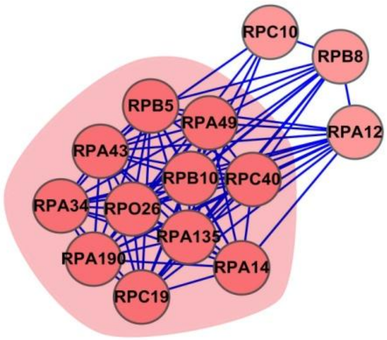

(h) RRW

Supplementary Figure 5: The RNA polymerase I complex as identified by the eight algorithms. Red nodes represent proteins that belong to the RNA polymerase I complex and white nodes represent proteins that do not belong to the RNA polymerase I complex. Shaded areas in each subgraph represent the clusters identified by a corresponding algorithm

The DASH complex was thoroughly examined in [4]. Among other seven methods, only ClusterONE is able to detect this complex completely and correctly[4]. ClusterEPs is also able to detect this complex completely and correctly. The result are shown in Supplementary Figure 6.

The RSC and the SWI/SNF complexes as a particular overlapping complex pair were also investigated in [4]. Both ClusterEPs and ClusterONE obtained a prediction result closest to the original RSC and the SWI/SNF complexes. The results are shown in Supplementary Figure 7. Other six methods are not able to achieve a good results[4].

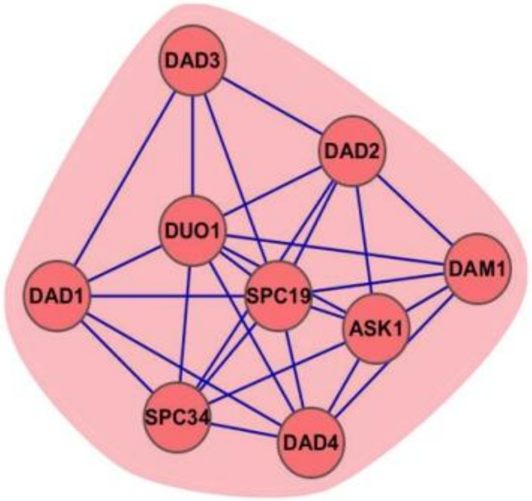

(a) ClusterONE

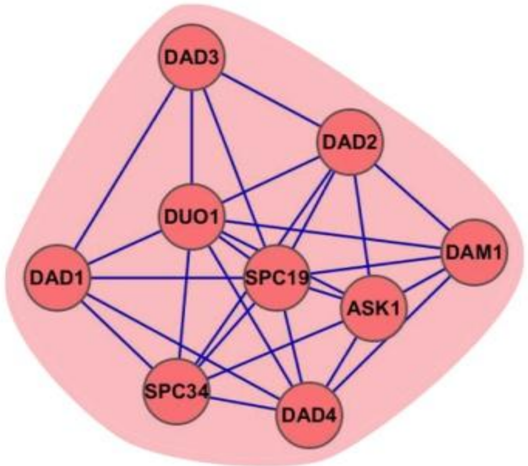

(b) ClusterEPs

Supplementary Figure 6: The DASH complex as identified by ClusterONE and ClusterEPs. Red nodes represent proteins that belong to the DASH complex. Shaded areas in each subgraph represent the clusters identified by the corresponding algorithm

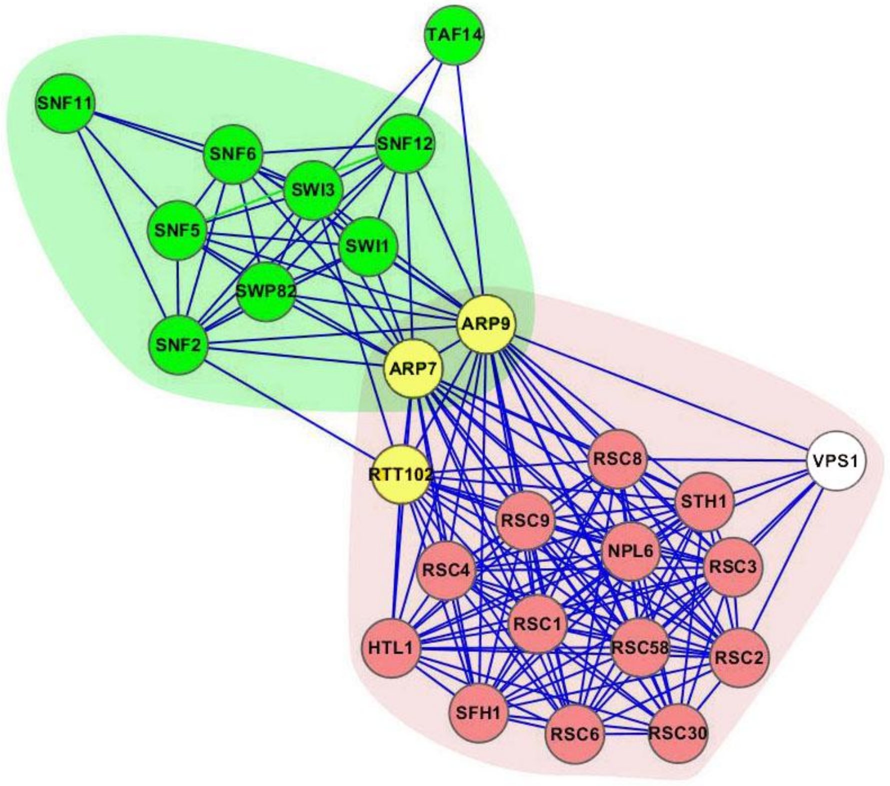

(a) ClusterONE

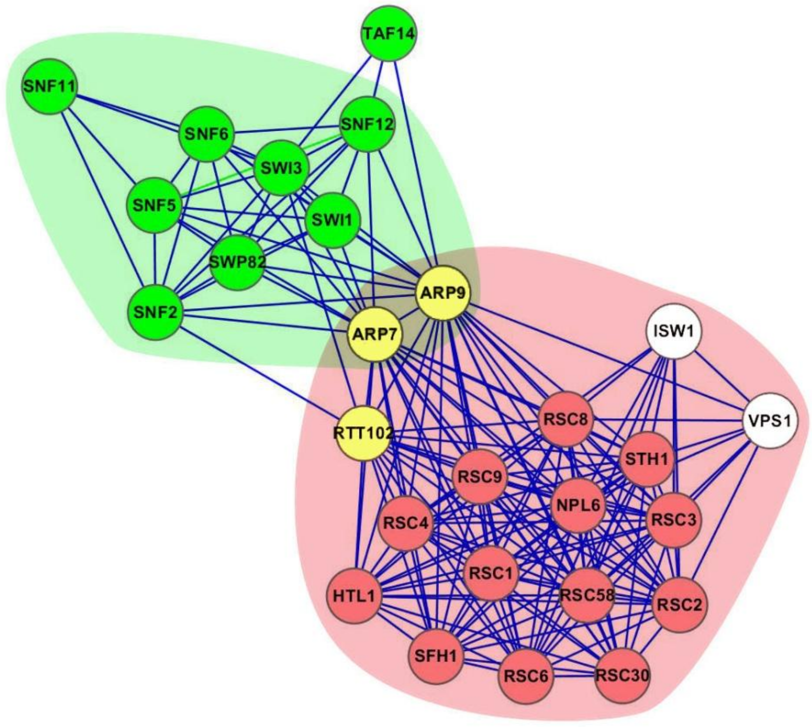

(b) ClusterEPs

Supplementary Figure 7: The RSC and SWI/SNF complexes as identified by ClusterONE and ClusterEPs. Red and green nodes represent proteins that belong to the RSC and SWI/SNF complex, respectively; yellow nodes belong to both complexes, white nodes belong to neither. Shaded areas in each subgraph represent the clusters identified by the corresponding algorithm.

9 Prediction of unknown but biologically interpretable complexes

9.1 Interpretable complexes

Supplementary Table 27 shows four examples of complexes identified by ClusterEPs. The third column shows other methods which can identify the corresponding complex. Three identified yeast complexes has not been characterized in MIPS and SGD. The identified human complexes has not been characterized in CORUM. HumanComplex_1 is detected by training yeast complexes on the integrated six yeast PPI. The features and values of these subgraphs are shown in **Supplementary Tables 28-31.**

Supplementary Table 27：Four examples of identified complexes

Complex	proteins	methods	datasets
YeastComplex-1	IMG2 MRPL4 MRP20 MRPL40 MRPL35 MRPL3 MRPL15 MRPL10 MRP7 MRPL28 MRPL17	ClusterEP	Krogan core
YeastComplex-2	DEP1 CTI6 SIN3 RXT2 SAP30 UME1 RPD3 PHO23 SDS3 UME6	ClusterEPs, CMC[8]	Krogan extended
YeastComplex-3	RPB5 RPC31 RPB8 RPB10 RPC82 RPC40 RPC37 RPC53 RET1 RPC19 RPO31 RPO26 RPC34 RPC25 RPC17 RPC11	ClusterEPs, CMC[8]	Collins
HumanComplex_1	TFE3 MITF SMAD3 FOS LEF1 CTNNB1 EP300 SMAD2 SMAD4	ClusterEP	HPRD

Supplementary Table 28 ： The feature vector of the YeastComplex-1

Feature	value	Feature	value	Feature	value
nodeSize	11	*maxDegreeCorrelation*	6.5	*eigenValue_2*	2.66
graphDensity	0.4	*meanClusteringCoeff*	0.47	*eigenValue_3*	2.29
meanDegree	4	*varClusteringCoeff*	0.29	*aveLength*	286.91
varDegree	2.36	*maxClusteringCoeff*	1	*maxLength*	390
medianDegree	3	*meanTopologicCoeff*	0.61	*aveWeight*	328.36
maxDegree	7	*varTopologicCoeff*	0.15	*maxWeigth*	439
meanDegreeCorrelation	4.81	*maxTopologicCoeff*	0.93		
varDegreeCorrelation	0.81	*eigenValue_1*	3.73		

Supplementary Table 29：The feature vector of the YeastComplex-2

Feature	value	Feature	value	Feature	value
nodeSize	10	*maxDegreeCorrelation*	8	*eigenValue_2*	1.79
graphDensity	0.76	*meanClusteringCoeff*	0.88	*eigenValue_3*	1.69
meanDegree	6.8	*varClusteringCoeff*	0.09	*aveLength*	546.4
varDegree	3.56	*maxClusteringCoeff*	1	*maxLength*	1536
medianDegree	7	*meanTopologicCoeff*	0.82	*aveWeight*	615.9
maxDegree	9	*varTopologicCoeff*	0.05	*maxWeigth*	1748
meanDegreeCorrelation	7.42	*maxTopologicCoeff*	0.89		
varDegreeCorrelation	0.43	*eigenValue_1*	6.41		

Supplementary Table 30：The feature vector of the YeastComplex-3

Feature	value	Feature	value	Feature	value
nodeSize	16	*maxDegreeCorrelation*	14.83	*eigenValue_2*	1.91
graphDensity	0.96	*meanClusteringCoeff*	0.96	*eigenValue_3*	1.59
meanDegree	14.38	*varClusteringCoeff*	0.02	*aveLength*	380.06
varDegree	0.98	*maxClusteringCoeff*	1	*maxLength*	1460
medianDegree	15	*meanTopologicCoeff*	0.96	*aveWeight*	428
maxDegree	15	*varTopologicCoeff*	0.01	*maxWeigth*	1623
meanDegreeCorrelation	14.46	*maxTopologicCoeff*	0.99		
varDegreeCorrelation	0.17	*eigenValue_1*	13.47		

Supplementary Table 31：The feature vector of the HumanComplex-1

Feature	value	Feature	value	Feature	value
nodeSize	9	*maxDegreeCorrelation*	5.8	*eigenValue_2*	1.84
graphDensity	0.58	*meanClusteringCoeff*	0.57	*eigenValue_3*	1.08
meanDegree	4.67	*varClusteringCoeff*	0.4	*aveLength*	723.67
varDegree	2.67	*maxClusteringCoeff*	1	*maxLength*	2414
medianDegree	5	*meanTopologicCoeff*	0.7	*aveWeight*	794
maxDegree	7	*varTopologicCoeff*	0.05	*maxWeigth*	2641
meanDegreeCorrelation	5.28	*maxTopologicCoeff*	0.75		
varDegreeCorrelation	0.6	*eigenValue_1*	3.92		

9.2 Gene ontology (GO) enrichment analysis

We have used BINGO [10] to conduct gene ontology (GO) enrichment analysis of five complexes (Supplementary Table 26) identified by ClusterEPs. The parameters and analysis methods are as follows:

(1) statistical test: Hypergeometric test;

(2) multiple testing correction: Benjamini & Hochberg False Discovery Rate(FDR) correction;

(3) significance level: 0.05;

(4) reference set: Use whole annotation as reference set;

(5) ontology file: GO-Biological_process.

(6) organism/annotation: Saccharomyces cerevisiae is selected for three yeast complexes, Homo sapiens is selected for two human complexes.

The analysis results for these four complexes are shown in S**upplementary Tables 32-36.**

Supplementary Table 32： GO Functional enrichment analysis for YeastComplex-1

GO ID	Description	p-value	genes
6412	translation	1.1386E-9	All 11 genes
34645	cellular macromolecule biosynthetic process	8.2494E-7	All 11 genes
9059	macromolecule biosynthetic process	8.6773E-7	All 11 genes
44267	cellular protein metabolic process	1.9023E-6	All 11 genes
10467	gene expression	2.4419E-6	All 11 genes
19538	protein metabolic process	3.4279E-6	All 11 genes
44249	cellular biosynthetic process	1.1566E-5	All 11 genes
9058	biosynthetic process	1.3618E-5	All 11 genes
44260	cellular macromolecule metabolic process	3.4741E-4	All 11 genes
43170	macromolecule metabolic process	4.5718E-4	All 11 genes
44238	primary metabolic process	3.3308E-3	All 11 genes
44237	cellular metabolic process	4.8593E-3	All 11 genes
8152	metabolic process	8.1061E-3	All 11 genes

Supplementary Table 33 GO Functional enrichment analysis for YeastComplex-2

GO ID	Description	p-value	genes
16575	histone deacetylation	3.6270E-25	All 10 genes
6476	protein amino acid deacetylation	1.2917E-24	All 10 genes
16569	covalent chromatin modification	6.0214E-19	All 10 genes
16570	histone modification	6.0214E-19	All 10 genes
45941	positive regulation of transcription	6.0243E-16	All 10 genes

10628	positive regulation of gene expression	6.3553E-16	All 10 genes
16568	chromatin modification	6.3553E-16	All 10 genes
45935	positive regulation of nucleobase, nucleoside, nucleotide and nucleic acid metabolic process	1.2426E-15	All 10 genes
51173	positive regulation of nitrogen compound metabolic process	1.2426E-15	All 10 genes
10557	positive regulation of macromolecule biosynthetic process	2.1200E-15	All 10 genes
31328	positive regulation of cellular biosynthetic process	2.9361E-15	All 10 genes
9891	positive regulation of biosynthetic process	2.9361E-15	All 10 genes
6325	chromatin organization	3.8498E-15	All 10 genes
10604	positive regulation of macromolecule metabolic process	6.2118E-15	All 10 genes
31325	positive regulation of cellular metabolic process	8.6760E-15	All 10 genes
9893	positive regulation of metabolic process	9.8063E-15	All 10 genes
48522	positive regulation of cellular process	2.8762E-14	All 10 genes
48518	positive regulation of biological process	5.0685E-14	All 10 genes
48523	negative regulation of cellular process	2.6308E-13	All 10 genes
48519	negative regulation of biological process	7.3388E-13	All 10 genes
51276	chromosome organization	1.3551E-12	All 10 genes
43687	post-translational protein modification	3.3331E-12	All 10 genes
6350	transcription	3.4979E-11	All 10 genes
6464	protein modification process	1.2529E-10	All 10 genes
45449	regulation of transcription	1.9886E-10	All 10 genes
43412	macromolecule modification	5.4140E-10	All 10 genes
19219	regulation of nucleobase, nucleoside, nucleotide and nucleic acid metabolic process	7.0826E-10	All 10 genes
51171	regulation of nitrogen compound metabolic process	7.2726E-10	All 10 genes
10468	regulation of gene expression	2.9262E-9	All 10 genes
10556	regulation of macromolecule biosynthetic process	3.4330E-9	All 10 genes
31326	regulation of cellular biosynthetic process	4.1538E-9	All 10 genes
9889	regulation of biosynthetic process	4.3903E-9	All 10 genes
60255	regulation of macromolecule metabolic process	1.1671E-8	All 10 genes
31323	regulation of cellular metabolic process	1.7621E-8	All 10 genes
80090	regulation of primary metabolic process	1.7621E-8	All 10 genes
19222	regulation of metabolic process	3.3791E-8	All 10 genes
6996	organelle organization	9.4284E-8	All 10 genes
50794	regulation of cellular process	3.3183E-7	All 10 genes
90304	nucleic acid metabolic process	4.9863E-7	All 10 genes
50789	regulation of biological process	8.9175E-7	All 10 genes
6139	nucleobase, nucleoside, nucleotide and nucleic acid metabolic process	2.2185E-6	All 10 genes
34645	cellular macromolecule biosynthetic process	2.9538E-6	All 10 genes
9059	macromolecule biosynthetic process	3.0927E-6	All 10 genes
65007	biological regulation	3.9675E-6	All 10 genes
16043	cellular component organization	4.0121E-6	All 10 genes
44267	cellular protein metabolic process	6.3120E-6	All 10 genes
10467	gene expression	7.9198E-6	All 10 genes
34641	cellular nitrogen compound metabolic process	7.9613E-6	All 10 genes

6807	nitrogen compound metabolic process	1.0299E-5	All 10 genes
19538	protein metabolic process	1.0779E-5	All 10 genes
44249	cellular biosynthetic process	3.2556E-5	All 10 genes
9058	biosynthetic process	3.7764E-5	All 10 genes
44260	cellular macromolecule metabolic process	7.1728E-4	All 10 genes
43170	macromolecule metabolic process	9.2060E-4	All 10 genes
44238	primary metabolic process	5.5977E-3	All 10 genes
44237	cellular metabolic process	7.8905E-3	All 10 genes

Supplementary Table 34： GO Functional enrichment analysis for YeastComplex-3

**GO ID**	**Description**	**p-value**	**genes**
6383	transcription from RNA polymerase III promoter	9.6854E-39	All 16 genes
6351	transcription, DNA-dependent	6.5360E-24	All 16 genes
32774	RNA biosynthetic process	7.5524E-24	All 16 genes
6350	transcription	1.7258E-17	All 16 genes
16070	RNA metabolic process	1.0550E-14	All 16 genes
90304	nucleic acid metabolic process	8.0639E-11	All 16 genes
6139	nucleobase, nucleoside, nucleotide and nucleic acid metabolic process	8.8268E-10	All 16 genes
34645	cellular macromolecule biosynthetic process	1.3965E-9	All 16 genes
9059	macromolecule biosynthetic process	1.5033E-9	All 16 genes
10467	gene expression	6.7847E-9	All 16 genes
34641	cellular nitrogen compound metabolic process	6.8417E-9	All 16 genes
6807	nitrogen compound metabolic process	1.0335E-8	All 16 genes
44249	cellular biosynthetic process	6.5349E-8	All 16 genes
9058	biosynthetic process	8.2888E-8	All 16 genes
44260	cellular macromolecule metabolic process	9.2607E-6	All 16 genes
43170	macromolecule metabolic process	1.3811E-5	All 16 genes`
44238	primary metabolic process	2.4869E-4	All 16 genes`
44237	cellular metabolic process	4.3092E-4	All 16 genes`
8152	metabolic process	9.0752E-4	All 16 genes`

Supplementary Table 35： GO Functional enrichment analysis for HumamComplex-1

**GO ID**	**Description**	**p-value**	**genes**
45944	positive regulation of transcription from RNA polymerase II promoter	7.2497E-15	All 9 genes
45893	positive regulation of transcription, DNA-dependent	7.2562E-14	All 9 genes
51254	positive regulation of RNA metabolic process	8.0856E-14	All 9 genes
45941	positive regulation of transcription	2.5770E-13	All 9 genes
10628	positive regulation of gene expression	3.9649E-13	All 9 genes
45935	positive regulation of nucleobase, nucleoside, nucleotide and	8.5041E-13	All 9 genes

	nucleic acid metabolic process		
10557	positive regulation of macromolecule biosynthetic process	1.1013E-12	All 9 genes
51173	positive regulation of nitrogen compound metabolic process	1.1312E-12	All 9 genes
31328	positive regulation of cellular biosynthetic process	1.9752E-12	All 9 genes
9891	positive regulation of biosynthetic process	2.2658E-12	All 9 genes
6357	regulation of transcription from RNA polymerase II promoter	2.7562E-12	All 9 genes
10604	positive regulation of macromolecule metabolic process	2.2237E-11	All 9 genes
31325	positive regulation of cellular metabolic process	2.8183E-11	All 9 genes
9893	positive regulation of metabolic process	4.5265E-11	All 9 genes
6355	regulation of transcription, DNA-dependent	8.0424E-9	All 9 genes
51252	regulation of RNA metabolic process	1.0238E-8	All 9 genes
48522	positive regulation of cellular process	2.0450E-8	All 9 genes
48518	positive regulation of biological process	4.9015E-8	All 9 genes
45449	regulation of transcription	2.3076E-7	All 9 genes
10556	regulation of macromolecule biosynthetic process	5.3266E-7	All 9 genes
10468	regulation of gene expression	6.2406E-7	All 9 genes
19219	regulation of nucleobase, nucleoside, nucleotide and nucleic acid metabolic process	8.1008E-7	All 9 genes
31326	regulation of cellular biosynthetic process	8.2719E-7	All 9 genes
51171	regulation of nitrogen compound metabolic process	8.7527E-7	All 9 genes
9889	regulation of biosynthetic process	8.8833E-7	All 9 genes
60255	regulation of macromolecule metabolic process	2.2572E-6	All 9 genes
80090	regulation of primary metabolic process	3.5767E-6	All 9 genes
31323	regulation of cellular metabolic process	5.6092E-6	All 9 genes
19222	regulation of metabolic process	8.6301E-6	All 9 genes
50794	regulation of cellular process	5.5660E-4	All 9 genes
50789	regulation of biological process	8.8634E-4	All 9 genes
65007	biological regulation	1.4897E-3	All 9 genes

References

[1] Qi, Y., Balem, F., Faloutsos, C., Klein-Seetharaman, J., and Bar-Joseph, Z. (2008). Protein complex identification by supervised graph local clustering. *Bioinformatics*, 24, i250–i268.

[2] Garcia-Borroto, M. (2010). Searching extended emerging patterns for supervised classification. Ph.D. thesis.

[3] García-Borroto, M. (2014). A survey of emerging patterns for supervised classification. *Artif Intell Rev.*, 42, 705–721.

[4] Nepusz, T. Yu, H. Paccanaro, A.(2012). Detecting overlapping protein complexes in protein-protein interaction networks. *Nat. Methods*, 9(5), 471-472.

[5] Palla, G., Derenyi, I., Farkas, I. & Vicsek, T. (2005). Uncovering the overlapping community structure of complex networks in nature and society. *Nature*, 435, 814-818.

[6] Adamcsek, B., Palla, G., Farkas, I., Der_enyi, I. & Vicsek, T. (2006). CFinder: locating cliques and overlapping modules in biological networks. *Bioinformatics*, 22, 1021-1023.

[7] King, A., Pr_zulj, N. and Jurisica, I. (2004). Protein complex prediction via cost-based clustering. *Bioinformatics*, 20, 3013-3020.

[8] Liu, G., Wong, L. and Chua, H. N. (2009). Complex discovery from weighted PPI networks. *Bioinformatics*, 25, 1891-1897.

[9] Bader, G. D. and Hogue, C. W. (2003). An automated method for finding molecular complexes in large protein interaction networks. *BMC Bioinformatics*, 4, 2.

[10] Maere, S., Heymans, K., and Kuiper, M.(2005). Bingo: a cytoscape plugin to assess overrepresentation of gene ontology categories in biological networks. *Bioinformatics*, 21, 3448–3449.
